# *Naja mossambica mossambica* Cobra Cardiotoxin Targets Mitochondria to Disrupt Mitochondrial Membrane Structure and Function

**DOI:** 10.3390/toxins11030152

**Published:** 2019-03-08

**Authors:** Boris Zhang, Feng Li, Zhengyao Chen, Indira H. Shrivastava, Edward S. Gasanoff, Ruben K. Dagda

**Affiliations:** 1 University of Nevada, Reno School of Medicine, Department of Pharmacology, Reno, NV 89557, USA; boris.zhang@wsu.edu; 2 STEM Program, Science Department, Chaoyang KaiWen Academy, North Dongba Road, Beijing 100016, China; Feng.Li@kaiwenedu.org (F.L.); 1806090003@cy.kaiwenacademy.cn (Z.C.); 3 Department of Computational and System Biology, University of Pittsburgh, PA, 15260, USA; ihs2@pitt.edu; 4 Department of Environmental and Occupational Health, University of Pittsburgh, PA,15260, USA

**Keywords:** cobra cardiotoxin, mitochondria, cardiolipin, mitochondrial dysfunction, non-bilayer membrane structures

## Abstract

Cobra venom cardiotoxins (CVCs) can translocate to mitochondria to promote apoptosis by eliciting mitochondrial dysfunction. However, the molecular mechanism(s) by which CVCs are selectively targeted to the mitochondrion to disrupt mitochondrial function remains to be elucidated. By studying cardiotoxin from *Naja mossambica mossambica* cobra (cardiotoxin VII4), a basic three-fingered S-type cardiotoxin, we hypothesized that cardiotoxin VII4 binds to cardiolipin (CL) in mitochondria to alter mitochondrial structure/function and promote neurotoxicity. By performing confocal analysis, we observed that red-fluorescently tagged cardiotoxin rapidly translocates to mitochondria in mouse primary cortical neurons and in human SH-SY5Y neuroblastoma cells to promote aberrant mitochondrial fragmentation, a decline in oxidative phosphorylation, and decreased energy production. In addition, by employing electron paramagnetic resonance (EPR) and protein nuclear magnetic resonance (^1^H-NMR) spectroscopy and phosphorescence quenching of erythrosine in model membranes, our compiled biophysical data show that cardiotoxin VII4 binds to anionic CL, but not to zwitterionic phosphatidylcholine (PC), to increase the permeability and formation of non-bilayer structures in CL-enriched membranes that biochemically mimic the outer and inner mitochondrial membranes. Finally, molecular dynamics simulations and in silico docking studies identified CL binding sites in cardiotoxin VII4 and revealed a molecular mechanism by which cardiotoxin VII4 interacts with CL and PC to bind and penetrate mitochondrial membranes.

## 1. Introduction

The ability of a variety of aggregated amyloid-like proteins to interact with lipid bilayers in organelles and cell membranes can occur under certain pathological conditions and in neurodegenerative diseases, which can lead to brain degeneration. For instance, in Parkinson’s disease (PD), neuronal dysfunction and degeneration in affected areas of the brain involves the aberrant folding and aggregation of the α-synuclein (αS) protein [1,2]. Although the precise physiological function of αS protein remains to be elucidated, several studies suggest that αS is involved in facilitating the assembly of large complexes at the presynaptic plasma membrane required for neurotransmission [3,4]. On the other hand, the aberrant folding of αS protein exposes hydrophobic domains on its surface, which promote self-assembly of αS into the stable toxic oligomeric structures that contain antiparallel β-sheets [5], one of the cardinal features in the neuropathology of PD [6]. The pathogenesis of PD largely relates to the ability of toxic oligomeric αS to disrupt the functions of cellular membranes [7], which include a generalized increase in membrane conductance, the formation of membrane channels and pores, and a disruption of the membrane structural integrity [8], including that of mitochondrial membranes (reviewed in [9]).

The molecular mechanisms by which αS binds to mitochondrial membranes have also been investigated. For instance, in dopaminergic cell lines and primary neurons that overexpress αS intracellularly, mitochondrial pathology (mitochondrial swelling, fission and loss of mitochondrial membrane potential, and release of cytochrome c), elevated levels of reactive oxygen species (ROS), an increase of mitochondrial Ca^2+^, subsequent bioenergetic failure, and a decrease in cell viability were observed [10,11,12]. Similar effects were also observed when neuronal cell lines were treated with oligomeric αS [10,12,13]. These effects are linked to the ability of αS oligomers to bind with cardiolipin (CL), an abundant phospholipid of the inner mitochondrial membrane (IMM), which results in the perturbation of the IMM structure [9]. Another consequence of the binding of oligomeric αS to CL is the loss of the function of the mitochondrial adenonsine diphosphate/ adenosine triphosphate (ADP/ATP) carrier, a membrane-bound protein, which is normally stabilized by CL molecules [14,15]. In addition, the association of oligomeric αS with CL leads to the formation of amyloid or lipidic pores within mitochondrial membranes [16]. Apart from oligomeric αS, other aggregated forms of amyloidogenic proteins, including amyloid beta (Aβ) and tau, can compromise the integrity of membranes that have a high amount of CL [17]. 

As amyloid-like proteins, cytotoxins and cardiotoxins derived from cobra venoms—which can form oligomeric proteins in a similar manner as amyloidogenic proteins [18]—can also interact with mitochondria by physically associating with CL in IMMs [19]. A previous study raised the possibility that amyloidogenic proteins and natural cytotoxins may share a common pore-forming mechanism involving the piercing of mitochondria via CL binding [9]. Cobra venom cardiotoxins (CVCs) have been used as a molecular tool to probe the structure and functions of biological membranes [20,21] by studying the structure of model membranes [22,23,24]. Importantly, CVCs have also been investigated as potential therapeutic agents to treat various diseases, including cancer [20,21,25,26,27]. Recently, CVCs have been described as potential therapeutic agents to restore mitochondrial bioenergetics in age-associated neurodegenerative diseases [19,20]. 

CVCs, also known as cardiotoxins or cytotoxins, are basic amphipathic proteins that contain a rigid spatial structure, including anti-parallel β-sheets (stabilized by four conserved disulfide bonds) and three elongated finger-like loops, each with apolar tips that are flanked by small stretches of highly conserved positively charged Lys and Arg residues (reviewed in [20]). In addition, cytotoxins have been classified as either S-type cytotoxins (contain Ser in residue position 28 of loop II) or as P-type cytotoxins (contain Pro in residue position 30 of loop II) [24]. As a rule, P-type cytotoxins have a higher net positive charge than S-type cytotoxins. P-type cytotoxins, having apolar Pro, but not polar Ser residues, have higher hydrophobicity in loop II compared to S-type cytotoxins, and this is believed to account for the higher cytotoxicity of P-type cytotoxins [24].

Although not widely known for their neurotoxicity, the pathophysiological effects of cytotoxins in neurons include their ability to promote neurotoxicity by depolarizing excitable membranes of neurons, deregulate the activity of cell membrane-bound enzymes and receptors, and thereby disrupt neurotransmission and promote axonal degeneration. Beyond the central nervous system, cytotoxins can inhibit platelet aggregation, and promote cardiac arrest (reviewed in [20]). These pathological effects are linked to the ability of cytotoxins to electrostatically interact with anionic lipids on the cell membrane to form stable oligomeric complexes that can act as pore-forming structures or to induce the formation of non-bilayer structures (reviewed in [20]). 

The molecular mechanisms by which CVCs promote cellular pathology remain to be elucidated. Recent evidence has shown that cytotoxins can target the membranes of intracellular organelles, such as lysosomes [26,28] and mitochondria [27,29]. For instance, *Naja naja atra* cardiotoxin 3 (CTX3) has been shown to target mitochondria to induce oxidative stress, leading to a collapse of the mitochondrial transmembrane potential, release of cytochrome C, and eventual activation of apoptosis [27]. 

We have shown that cytotoxins have the ability to remodel the lipid membranes of mitochondria to modulate mitochondrial bioenergetics. By employing a battery of biophysical, biochemical, and computer modeling assays, our recently published studies showed that two cytotoxins from *Naja naja oxiana* cobra venom bind specifically to CL in model membranes and induce the formation of non-bilayer structures in membranes in intact mitochondria (reviewed in [20]). Additionally, we show that the same two cytotoxins (CTI and CTII) induce the formation of a transient non-bilayer phase in mitochondrial membranes at very low concentrations, a phenomenon that leads to increased ATP synthase activity [30,31]. This observation suggests that the formation of transient non-bilayer structures is a physiological event that occurs to support the proper structure and function of mitochondria [30,31]. Interestingly, higher concentrations of cytotoxins induced a significant amount of a non-bilayer phase in IMMs, which surpassed that of the lamellar phase and completely abolished ATP synthase activity, which was due to the ability of the cytotoxin to disrupt the IMM [30,31]. However, the molecular mechanism by which S-type cardiotoxins can bind to mitochondrial membranes to elicit mitochondrial dysfunction in cells has not been elucidated.

The molecular surface features of amyloidegenic proteins and cobra cardiotoxins are particularly similar—both proteins have a positively charged N-terminal region, contain a central region with predominantly hydrophobic residues having a high propensity for adopting the β-sheet secondary structure, and harbor acidic residue(s) in the C-terminal domain—all of which likely underlie their shared membrane-active properties, such as the formation of transmembrane pores, disruption of membrane packing, and targeting of mitochondrial CL [8,9,12,30,31]. Given these shared membrane-active properties, we hypothesized that cardiotoxin VII4 (CTX3) from *Naja mossambica mossambica*, similarly to amyloidegenic proteins, promotes neurotoxicity by translocating mitochondria to disrupt mitochondrial function via a mechanism that involves binding to CL. In this study, we show that cardiotoxin VII4, an S-type cardiotoxin that possesses an unusually high positive charge, translocates to mitochondria in mouse primary cortical neurons and in SH-SY5Y neuroblastoma cells to promote aberrant mitochondrial fragmentation, a reduction in oxidative phosphorylation, and overt neurodegeneration. By utilizing EPR and ^1^H-NMR spectroscopy and phosphorescence quenching of erythrosine in model membranes, we further show that cardiotoxin VII4 binds to anionic CL, but not to zwitterionic phosphatidylcholine (PC), to induce the formation of non-bilayer structures in CL enriched membranes that model the outer mitochondrial membrane (OMM) and inner mitochondrial membrane (IMM). Finally, molecular dynamics and in silico docking studies revealed the molecular mechanism by which cardiotoxin from *Naja mossambica mossambica* interacts with CL to intercalate into mitochondrial membranes.

## 2. Results

### 2.1. Cardiotoxin VII4 Promotes Cell Death in a Concentration Dependent-Manner

**Figure 1:**
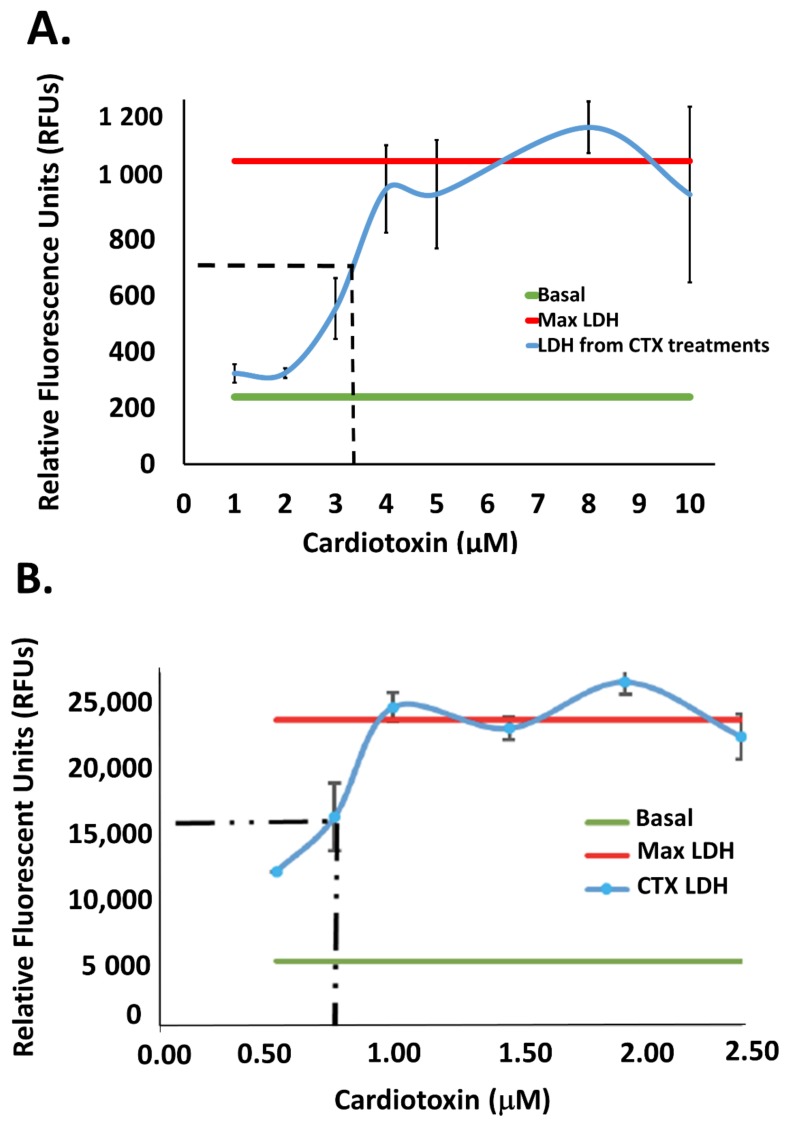
Cardiotoxin VII4 elicits neurodegeneration in a concentration-dependent manner. Exposure of cardiotoxin induces an increase in the release of lactate dehydrogenase (LDH), a measure of cell death predominantly induced by necrosis. The basal level (green line) shows the cellular level of LDH released in the medium without VII4 treatment (approximately 15%–17% death), whereas the maximum LDH level (red line) is measured by treating cells with 0.01% Triton X-100 (red line). The LD_50_, as demarcated by the halfway point between the basal and maximum LDH release (dashed lines), is induced at (**A**) 3.5 μM in SHSY5Y neuroblastoma and (**B**) at 0.75 μM for primary cortical neurons, respectively.

Cardiotoxins can induce neuropathology in the affected victim by interfering with axonal conduction and cytotoxity (reviewed in [20]). However, the molecular mechanisms by which cardiotoxins can promote neurotoxicity remain to be elucidated. Given its similar biophysical properties as amyloid-like proteins known to target mitochondria (e.g., α-synuclein), we hypothesize that cardiotoxin VII4 can promote mitochondrial dysfunction by binding to anionic phospholipids [7,17]. For this study, we employed both primary cortical neurons and human neuroblastoma SH-SY5Y cells to study mitochondrial pathology and neurodegeneration induced by cardiotoxin VII4. In order to characterize the neurotoxicity of cardiotoxin from *Naja mossambica mossambica* (cardiotoxin VII4), we performed several survival assays by performing the lactate dehydrogenase (LDH) release assay to identify the lethal dose (LD_50_) in primary cortical neurons and in SH-SY5Y neuroblastoma cells treated with increasing concentrations of cardiotoxin. Following three representative experiments, we observed that the LD_50_s for cardiotoxin VII4 is 3.5 μM for primary cortical neurons and 0.75 μM in SH-SY5Y cells. In addition, the sigmoidal shape of each LDH curve suggests that the range by which VII4 can promote cytotoxicity in neuroblastoma cells falls within of 1–4 µM (Figure 1A) and 0.5–1 µM for primary cortical neurons (Figure 1B), respectively.

### 2.2. Bioenergetic Alterations in SH-SY5Y Neuroblastoma Following Treatment with Cardiotoxin VII4

**Figure 2:**
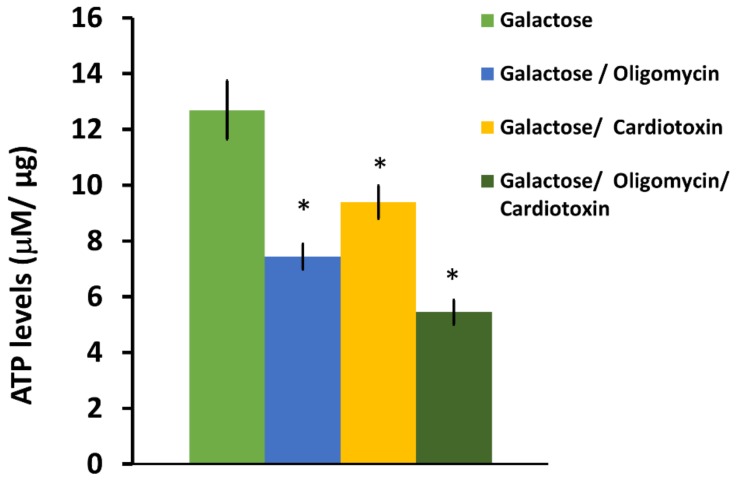
Cardiotoxin VII4 promotes a loss in mitochondrial-derived ATP levels. Graph showing the mean level of ATP (µM) per μg of protein as measured by using luciferase-based assays in untreated or VII4-treated neuroblastoma cells grown in media containing galactose in the presence or absence of oligomycin in order to determine the fraction of ATP derived from mitochondria and from glycolysis. Following exposure of SH-SY5Y neuroblastoma to VII4 (4 h at the LD_50_), a significant decrease in total ATP production (green bar vs. yellow bar) and of mitochondrial-derived ATP levels (galactose—galactose/oligomycin; galactose/cardiotoxin—galactose/oligomycin/cardiotoxin) suggesting that cardiotoxin VII4 significantly reduced ATP levels derived from glycolysis and mitochondria. (*: *p* < 0.05 vs. galactose, n = 8 wells of neurons analyzed per experiment, representative experiment of three assays, one-way ANOVA with Tukey’s test).

In order to analyze the effects of cardiotoxin VII4 on energy production in primary cortical neurons, we performed luminescence-based ATP assays and measured mitochondrial respiration by employing an XF^e^24 Flux Analyzer in cells treated with an LD_50_ of cardiotoxin VII4. Following the exposure of SH-SY5Y neuroblastoma cells with an LD_50_ of VII4 (3.5 μM) for 4 h, we observed that the total ATP levels dropped significantly in neuroblastoma cells (Figure 2). In addition, treating primary cortical neurons with VII4 causes significant decreased levels in mitochondrial-derived ATP as determined by treating cells with the ATP synthase inhibitor, oligomycin, for 30 min. Overall, our ATP assays suggest that acute exposure of neurons with VII4 promotes a 25.98% decrease in total ATP levels. This significant decrease in total ATP levels includes a 10.91% decrease in mitochondrial-derived ATP and a 14.81% in glycolysis-derived ATP in cardiotoxin-treated cells compared to untreated cells. The observation suggests that cardiotoxin VII4 is able to elicit a significant decrease in mitochondrial-derived ATP levels, an indication of impaired mitochondrial function.

### 2.3. Cardiotoxin VII4 Translocates to Mitochondria in Primary Neurons and SH-SY5Y Cells

**Figure 3:**
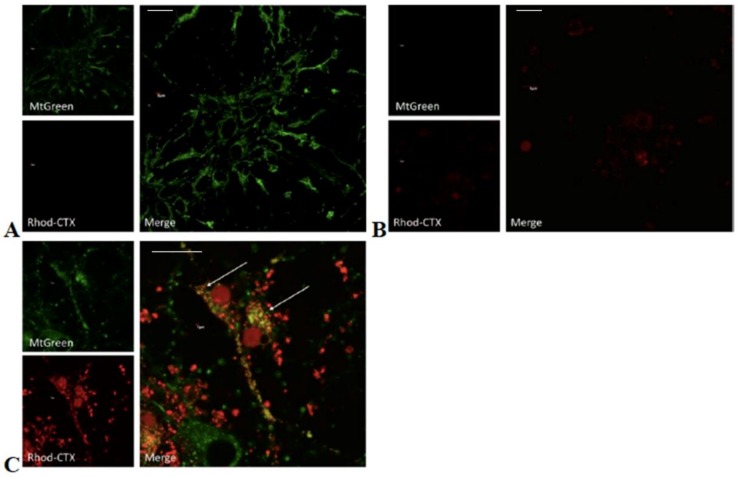
VII4 translocates to mitochondria and induces mitochondrial fragmentation in primary cortical neurons. Primary cortical neurons show colocalization of Rhodamine-cardiotoxin VII4 (red) with mitochondria (green) within 3 h of treatment **A**. Control using MitoTracker Green FM to label mitochondria. **B**. Control using Rhodamine-cardiotoxin VII4 to show cellular uptake. **C**. Neurodegeneration is shown by mitochondrial fragmentation and disintegration of neurites. Arrows show the presence of colocalizing structures (yellow). Scale bar: 20 μm.

**Figure 4:**
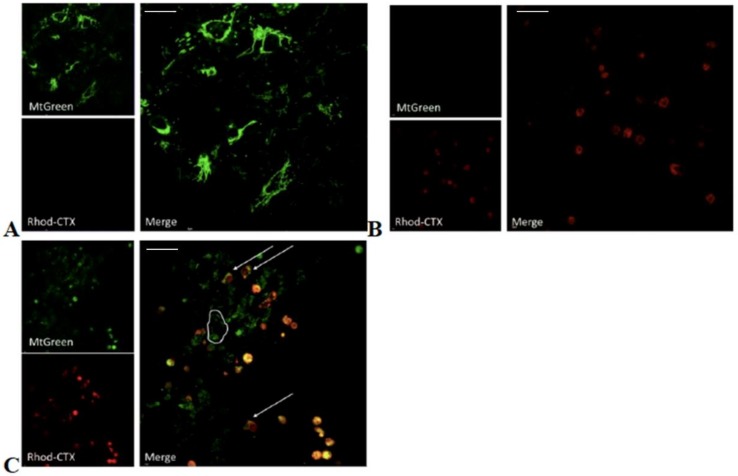
VII4 translocates to mitochondria and induces mitochondrial fragmentation in SH-SY5Y cells. **A**. Representative confocal micrographs demonstrating that in the absence of cardiotoxin VII4, SH-SY5Y cells predominantly contained tubular mitochondrial networks—an indication of healthy neuroblastoma cells as visualized by exposing cells to mitochondrial specific, fluorescent dye MitoTrackerGreen FM dye. **B**. Red fluorescent puncta within neuronal cells indicate cellular uptake of VII4-Rhodamine within 0.5 h of treatment. **C**. For clarity, an outline is drawn around the cell to show a neuroblastoma that did not uptake VII4-Rhodamine. The arrows indicate cells that have taken up cardiotoxin VII4-Rhodamine. Scale bar: 50 μm.

Given that VII4 can reduce the levels of ATP derived from the ATP synthase (Figure 2), we hypothesize that VII4 can translocate to mitochondria to promote mitochondrial dysfunction. In support of this concept, cardiotoxin VII4 contains a high number of lysine residues and an overall positive charge, which raises the possibility that VII4 can directly interact with negatively charged mitochondrial membranes in a similar manner as reported for CTI and CTII from *Naja naja oxiana* [19]. In order to address this hypothesis, we fluorescently labeled cardiotoxin VII4 with the red fluorescent conjugate, rhodamine, to track the localization of cardiotoxin in live primary cortical neurons and in neuronal cells by employing confocal microscopy. In brief, primary cortical neurons and SH-SY5Y neuroblastoma were treated with Mitotracker Green to visualize mitochondria (100 nM) prior to exposure to an LD_50_ (1.3 µM) of rhodamine conjugated cardiotoxin VII4 (cardiotoxin VII4-Rhodamine). The colocalization of cardiotoxin VII4-Rhodamine with MitoTracker Green FM-labeled mitochondria was visualized in a time-dependent manner by employing an Olympus Fluoview 1000 confocal microscope. Controls for the mitochondrial colocalization assays included primary cortical neurons or SH-SY5Y neuroblastoma stained with MitoTracker Green FM (Life Technologies) alone, cells exposed to rhodamine conjugate only, unlabeled cardiotoxin VII4, or cells exposed to cardiotoxin VII4 stained with rhodamine only. For all aforementioned control conditions, we did not detect non-specific background or fluorescence bleed-over across channels (e.g., Rhodamine conjugate bleeding over the green channel, or MitoTracker Green bleeding over the red channel). Interestingly, within the 30 min of exposure, we observed that a significant proportion of primary cortical neurons (>50%) had taken up the cardiotoxin, VII4-Rhodamine, and showed visible colocalization with mitochondria (Figure 3). Similar results were observed in SH-SY5Y neuroblastoma cells treated with cardiotoxin VII4-Rhodamine (Figure 4). 

**Figure 5:**
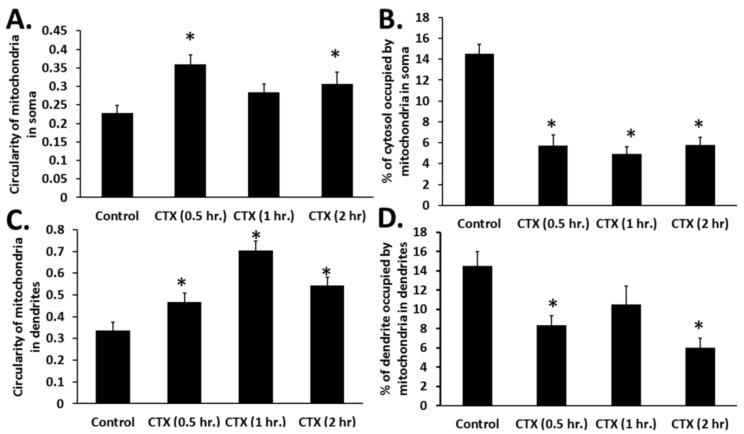
VII4 significantly fragments mitochondria and decreases mitochondrial levels in neurons. Time-dependent image analysis-mediated quantification of mitochondrial circularity (**A**–**C**), an index of mitochondrial fragmentation, and of mitochondrial content in the soma (**B**) or dendrites (**D**) was determined in fluorescent images of primary neurons treated with VII4 (CTX) or with vehicle control (control) at the indicated time points. The data shows that treatment of primary cortical neurons with VII4 significantly promotes mitochondrial fragmentation (increased circularity) and loss of mitochondrial content by 0.5 h of treatment. (For A–D. *: *p* < 0.05 vs. control, n = 15–30 neurons analyzed per experiment, representative experiment of two assays, one-way ANOVA with Tukey’s test).

To quantify the morphology of mitochondria in primary cortical neurons treated with cardiotoxin VII4-Rhodamine, we performed semi-automated image analysis of the ratio of area/perimeter and circularity (mitochondrial fragmentation) as previously published [32]. In brief, in untreated primary cortical neurons, we observed that mitochondria within the dendrites of neurons treated with cardiotoxin VII4 showed a significant increase in the average mitochondrial circularity and a concomitant reduction in mitochondrial content (% of soma or dendrite occupied by mitochondria) in the soma and dendrites (Figure 5). Interestingly, time-dependent analysis of mitochondrial morphology showed that the exposure of neurons to VII4 can rapidly fragment mitochondria, elicit significant loss of mitochondria within 30 min (Figure 5), and promote significant swelling of mitochondria at later time points (2 h, data not shown). As a specificity control, we observed that exposing primary cortical neurons to NHS-Rhodamine conjugate in media alone for 2–4 h did not permeate live cells nor did it translocate to the mitochondria as evident by the lack of colocalization with MitoTracker Green. The NHS-Rhodamine alone did not cause neurotoxicity as neurons treated with NHS-Rhodamine were completely adherent and showed no signs of degeneration following a 24 h exposure of the dye (data not shown). Overall, these control experiments suggest that cardiotoxin VII4, and not NHS-Rhodamine, is the targeting moiety for translocating cardiotoxin VII4-Rhodamine to the mitochondrion. Unexpectedly, we observed that cardiotoxin VII4-Rhodamine colocalized with nuclei in both the primary neurons and SH-SY5Y neuroblastoma cells (Figure 3 and Figure 4). However, this phenomenon is likely an artifact given that we observed that treatment of primary cortical neurons with rhodamine conjugate alone showed noticeable translocation of rhodamine to the nucleus only in primary neurons that have a necrotic or apoptotic appearance, suggesting that the NHS-Rhodamine conjugate translocates to the nuclei of dyeing neurons in a similar manner as propidium iodide.

Overall, our fluorescent imaged-based data suggest that cardiotoxin VII4 elicits rapid alterations in mitochondrial structure. 

### 2.4. Cardiotoxin VII4 Promotes Mitochondrial Pathology

**Figure 6:**
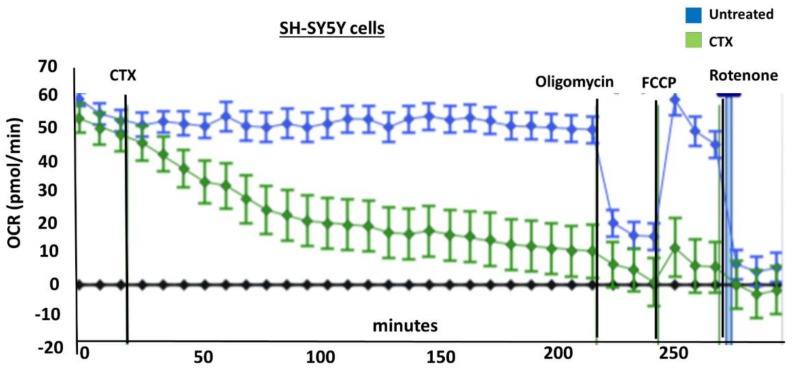
Treating neuroblastoma cells with cardiotoxin VII4 progressively decreases mitochondrial function. Oxygraph (oxygen consumption trace) acquired by using an XF24 Flux Analyzer demonstrates that SH-SY5Y cells treated with an LD_50_ of cardiotoxin VII4 (CTX, 20 green trace), significantly decreases baseline oxygen consumption rates (OCRs), ATP-dependent OCRs (oligomycin), maximal OCRs (Carbonyl cyanide-p-trifluoromethoxyphenylhydrazone FCCP), and mitochondrial-dependent OCRs (rotenone) within 15 min of cardiotoxin exposure while untreated cells (blue trace) are not affected. The oxygraph shown on this figure is representative of two experiments that showed similar results.

Previous studies have shown that CVCs can be targeted to the mitochondrion to reduce the mitochondrial membrane potential and promote mitochondrial fragmentation [27,29]. These published observations suggest that CVCs can selectively impair the mitochondrial membrane. To this end, we surmised that aberrations in mitochondrial morphology caused by exposure to cardiotoxin VII4 is associated with reduced mitochondrial function. By employing a XF^e^24 Extracellular Flux Analyzer to measure mitochondrial respiration, we observed that cardiotoxin VII4-treated primary cortical neurons showed progressively reduced baseline oxygen consumption rates (OCRs) within 30 min of exposure and significantly showed up to 80% of reduced baseline OCRs by 3 h of treatment (Figure 6). The significant decrease in OCRs within 30 min of treatment is consistent with our mitochondrial morphology assays in that VII4 significantly promotes robust mitochondrial fragmentation and loss of mitochondria (Figure 5). 

**Figure 7:**
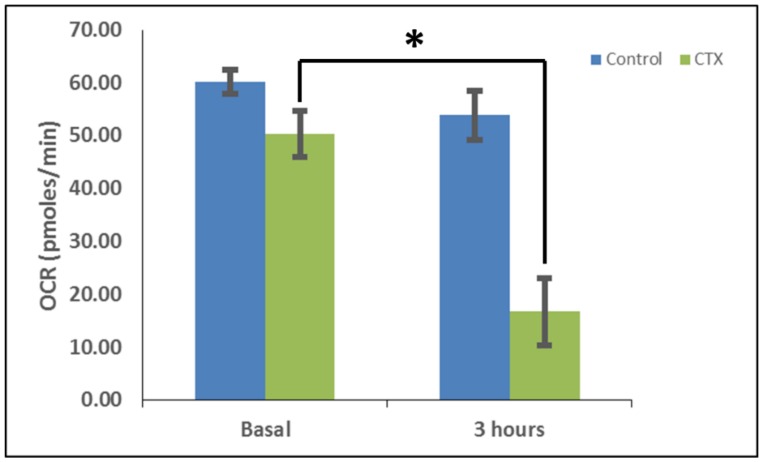
Cardiotoxin VII4 induces a significant reduction in baseline mitochondrial respiration. By employing a SeaHorse Bioscience XF^e^24 Analyzer, the OCRs were analyzed for up to three hrs, a time point for which little cell death is induced by VII4 (data not shown). The control had a simple injection of XF media while the second group of SH-SY5Y neuroblastoma cells was injected with the LD_50_ concentration of VII4, which resulted in a significant decrease of OCR over three hours. (*: *p* < 0.05 vs. basal, n = 8 wells of culture primary cortical neurons analyzed per experiment, representative experiment of two assays, one-way ANOVA with Tukey’s test).

Furthermore, in order to determine the extent to which cardiotoxin VII4 affects the respiratory spare capacity, neuroblastoma SH-SY5Y cells were treated with the mitochondrial uncoupler, Carbonyl cyanide-p-trifluoromethoxyphenylhydrazone (FCCP), to permeabilize the IMM, which thereby destroys the proton gradient at the IMM and elicits maximal OCRs. The difference between the maximal and baseline OCRs is defined as the spare respiratory capacity (spare respiratory capacity = FCCP-induced maximal OCR − basal OCR) [33]. Following an injection of FCCP (Figure 6, 250 min), we observed that SH-SY5Y neuronal cells treated with cardiotoxin VII4 contained a significantly lower maximal OCRs compared to untreated neurons (~80% reduction) and reduced the mitochondrial reserve capacity compared to untreated cells (Figure 7, FCCP at 250 min). 

### 2.5. Cardiotoxin VII4 Binds to Lipid Bilayers Enriched with CL

Given that cytotoxins can interact with CL and the fact that cardiotoxin VII4 binds to mitochondria to promote mitochondrial dysfunction (Figure 3, Figure 4, Figure 5, Figure 6 and Figure 7), we surmised that cardiotoxin VII4 specifically interacts with CL in mitochondrial membranes. To address this hypothesis, we performed a battery of biophysical experiments (phosphorescence quenching assays, electron paramagnetic resonance studies, membrane permeability assays) described below to test the mechanism by which cardiotoxin VII4 may interact with lipid bilayers that contain a similar lipid composition (PC + Cl) as found in the OMM and the IMM. To this end, we prepared unilamellar liposomes containing a fixed molar amount of PC and increasing concentrations of CL (including PC liposomes containing either 5% CL or 20% CL, lipid compositions that simulate the OMM and the IMM, respectively [9]) to study the ability of cardiotoxin VII4 to interact with CL. We employed erythrosine phosphorescence quenching by ferrocene to determine the ability of cardiotoxin VII4 to interact with unilamellar liposomes. The lifetime of a luminescent probe depends on the rate of clashes between a luminescent probe and its quencher. For this particular biophysical assay, it is important to note that erythrosine is located on the edge of lipid bilayers, specifically between polar heads and the hydrophobic phase of alkyl chains, whereas ferrocene is located within a hydrophobic phase consisting of alkyl chains. Hence, a decrease in the lateral and rotational movement of lipids in a bilayer and/or of the transition of a bilayer phase into non-bilayer structures is expected to increase the lifetime of erythrosine quenched by ferrocene [19,21], presumably as a result of a decrease in the lateral movement of lipids and by the formation of non-bilayers, which can hinder the rate of clashes between erythrosine and ferrocene. In our study, we observed no change in the lifetime of erythrosine phosphorescence in pure PC liposomes exposed to increasing concentrations of cardiotoxin VII4 (Table 1), suggesting that cardiotoxin does not interact with lipid bilayers that consist of merely PC. On the other hand, in PC liposomes containing CL, the lifetime of erythrosine phosphorescence increased in response to an increase in CL content in PC liposomes treated with increasing concentrations of cardiotoxin VII4 (Table 1). Hence, our data show that cardiotoxin binds to CL-containing membranes and reduces the lateral and rotational mobility of phospholipids, presumably due to the formation of non-bilayer structures.

### 2.6. Cardiotoxin VII4 Interacts CL-Enriched Cipid Bilayers to Induce the Formation of Non-Bilayer Structures

**Figure 8:**
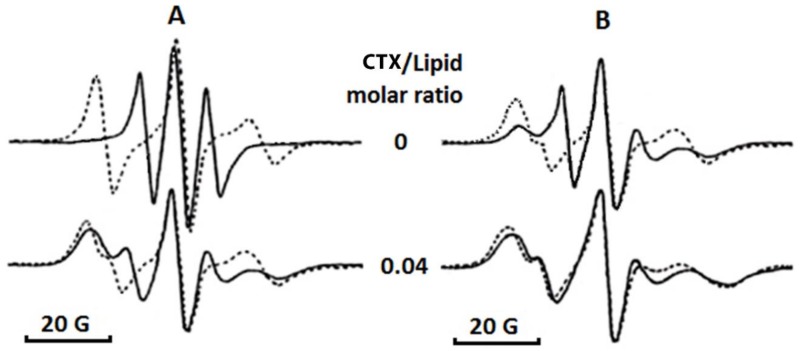
EPR spectra of 5-doxyl stearic acid ( 5-DSA) in oriented multibilayer membrane films of PC + 5 mol% CL (**A**) and in multibilayer membrane films of PC + 20 mo% CL (**B**) containing 5-DSA: lipid molar ratio 1:100 at 18 °C. The EPR spectra in multibilayer films were recorded with the magnetic field parallel (broken lines) and perpendicular (solid lines) to the bilayer normal. Multibilayer films were treated with cardiotoxin VII4 at the indicated cardiotoxin to lipid molar ratios.

Next, we employed electron paramagnetic resonance (EPR) studies of 5-doxyl stearic acid spin probe (5-DSA) in oriented multibilayer films as a sensitive biophysical method to analyze for changes in the angular distribution of the long molecular axes of membrane lipids and for changes in the lateral and rotational movement of lipids. In the absence of cardiotoxin, lipid bilayers, as modeled by multibilayer films, show strong anisotropy (non-superimposed EPR spectra at two angular orientations, Figure 8). In addition, Figure 8 shows that cardiotoxin decreases the angular anisotropy of long molecular axes of 5-DSA in PC + CL films, as observed by the superimposition of the shapes of EPR spectra taken at parallel and perpendicular orientations in PC + CL films containing cardiotoxin compared to non-superimposed EPR spectra observed in cardiotoxin-free phospholipid films in the applied magnetic field. It should be noted that in PC films containing 5% CL, cardiotoxin potentiated a decrease in the angular anisotropy of 5-DSA to a lesser extent compared to PC films containing 20% CL, as evident by a complete superimposition of the EPR spectra taken from parallel and perpendicular orientations (Figure 8).

Furthermore, to quantify for morphological changes in the EPR spectra of 5-DSA in multibilayer membranes treated with cardiotoxin VII4, we analyzed the *B*/*C* and *S* parameters (Table 2) as previously published [21,22]. A decrease in the value of the *B*/*C* parameter is indicative of the formation of non-bilayer structures whereas an increase in the parameter *S* value is due to a decrease in the molecular mobility of 5-DSA. These are well-characterized biophysical parameters that are altered by the cobra venom cytotoxins upon binding to anionic phospholipids [21,22]. As observed in Table 2, cardiotoxin VII4 does not bind to pure PC membranes as indicated by a lack of changes in the *B*/*C* and *S* values. However, cardiotoxin induced the formation of non-bilayer structures and a decrease in the molecular mobility of 5-DSA in PC membranes containing CL, presumably as a result of cardiotoxin binding to these membranes. In addition, the biophysical effects of cardiotoxin were more accentuated in PC films containing 20 mol% CL relative to PC films containing 5 mol% CL (Table 2).

### 2.7. Cardiotoxin VII4 Interacts with Liposomes Containing CL to Increase Membrane Permeability

**Figure 9:**
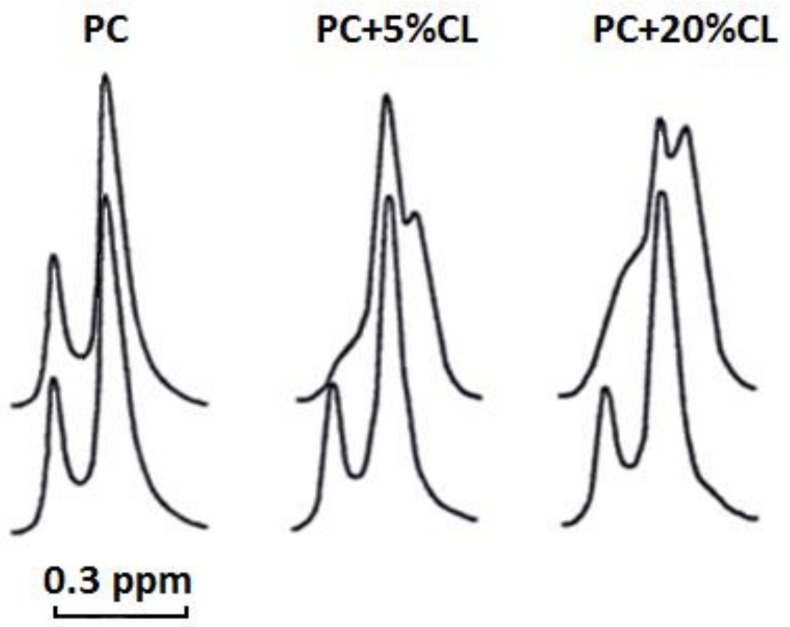
^1^H-NMR signals of N^+^(CH_3_)_3_ groups of PC of unimallemar liposomes in the presence of shift reagent, [Fe(CN)_6_]^−3^. The ^1^H-NMR signals shown at the bottom represent liposomes in the absence of cardiotoxin while the upper set of ^1^H-NMR signals represent liposomes in the presence of 2.1 × 10^−4^ M cardiotoxin. The total concentration of phospholipids in unilamellar liposomes is 1.4 × 10^−2^ M. The composition of the liposomes is indicated for each group of cardiotoxin treated and untreated liposomes.

Next, to investigate the extent to which cardiotoxin VII4 can affect the structural integrity of PC membranes containing CL, we performed ^1^H-NMR spectroscopy analysis of choline groups, (N^+^(CH_3_)_3_), in PC liposomes containing paramagnetic ferricyanide anion, [Fe(CN)_6_]^−3^, that was added into the solution on the outer leaflet side of the liposomes. While ferricyanide anion shifts the ^1^H-NMR signal of the outer leaflet choline groups toward a stronger field, it is unable to permeate lipid membranes and therefore does not reach the inner leaflet choline groups. Hence, the position of the ^1^H-NMR signal of the inner leaflet N^+^(CH_3_)_3_ groups is not affected and yields separate ^1^H-NMR signals for both the inner and outer leaflets N^+^(CH_3_)_3_ groups (Figure 9—lower set of signals). In addition, we observed that cardiotoxin has no effect on the ^1^H-NMR signals of pure PC liposomes, suggesting that cardiotoxin does not bind to PC membrane and thereby is unable to permeabilize membranes consisting of just PC. However, the addition of cardiotoxin to liposomes containing CL resulted in a noticeable shift of the ^1^H-NMR signal from the inner leaflet toward the stronger field (Figure 9—upper set of signals), suggesting that CL-enriched liposomes became permeable to ferricyanide anions. In addition, a new resonance signal appeared as a high-field peak on the outer leaflet signal in liposomes containing CL, which was more intense in liposomes containing 20 mol% of CL compared to liposomes containing 5 mol% CL. Hence, this new shift in the resonance frequency toward an even stronger field reflects the change in the interaction between the ferricyanide anions and N^+^(CH_3_)_3_ groups of the outer leaflet (Figure 9), presumably due to the formation of nonbilayer structures within the lipid bilayer of liposomes as previously described [19,21]. 

Overall, our collective biophysical data (EPR, erythrosine phosphorescence, and ^1^H-NMR) conclusively show that cardiotoxin specifically and avidly binds to phospholipid membranes containing CL (Figure 7, Figure 8 and Figure 9, Table 2). In addition, our biophysical data suggests that CL is involved in the formation of non-bilayer structures in lipid bilayers of a similar phospholipid composition as that found in mitochondrial membranes.

### 2.8. Molecular Dynamics Shows that Cardiotoxin Electrostatically Interacts with CL at the OMM

**Figure 10:**
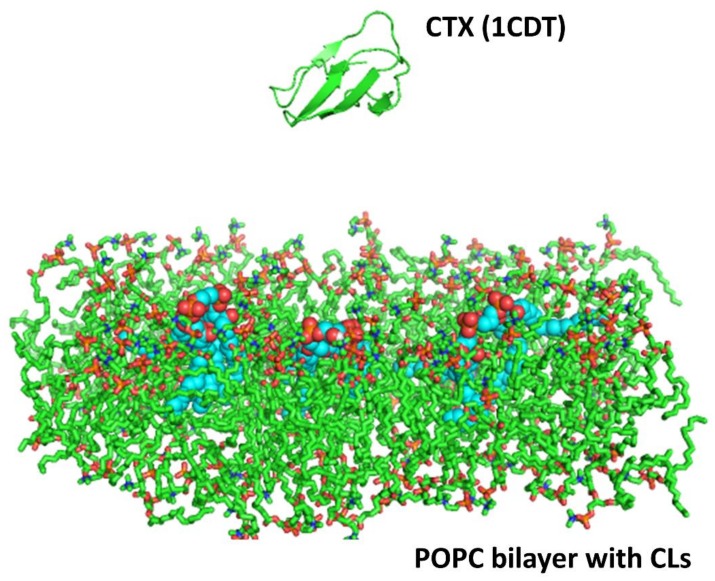
Set up of the molecular dynamic runs. The interaction of cardiotoxin (cardiotoxin VII4 PDB ID: 1CDT) with an in silico lipid bilayer was analyzed by performing molecular dynamics runs. For each molecular dynamics run, the system consisted of the crystal structure of cardiotoxin VII4 (CTX, PDB ID: 1CDT, shown as a ribbon diagram of β-strands and loops), which was positioned approximately 20 Å away from a phospholipid bilayer that consisted of 128 POPC molecular with or without three cardiolipin (CL) molecules. Note that the POPC bilayer containing CLs is of a similar phospholipid composition similar to that of the outer mitochondrial membrane (OMM). In the figure, the cardiolipin molecules are shown in cyan, space-filling representation.

**Figure 11:**
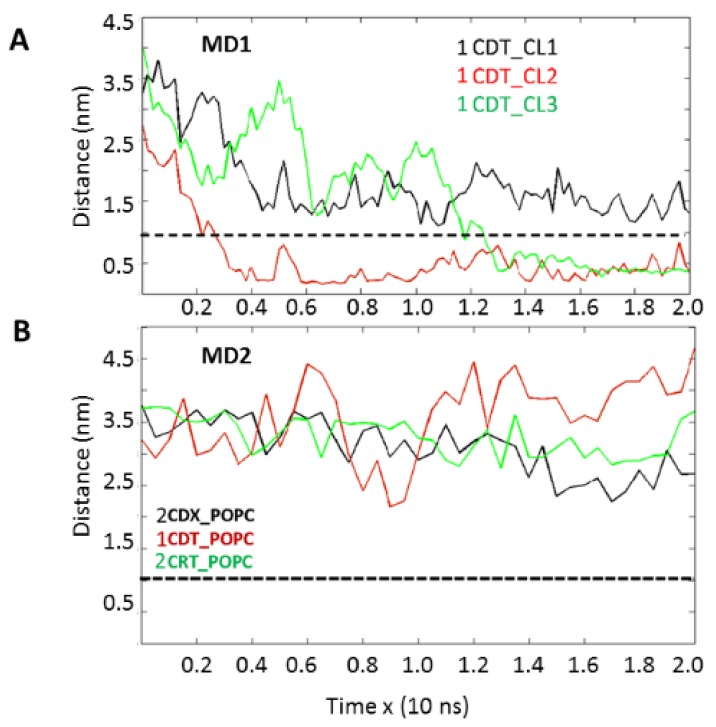
Cardiotoxin VII4 is electrostatically attracted to CL in POPC bilayers. **A**. Time evolution analysis of molecular dynamic runs of 10 nanoseconds suggest that cardiotoxin VII4 interacts with up to two CL molecules in POPC bilayers containing CL as shown by the ability of cardioxotin VII4 (1CDT) to reach the threshold (dashed line) for generating inter-molecular interactions. **B**. Time evolution analysis of molecular dynamic runs showed that cardiotoxin VII4 (1CDT) or other cardiotoxin from other snake venom species (2CDT and 2CRT) is unable to interact with the POPC bilayer that lacked CL as evident by their inability to reach the threshold (dashed line) for generating inter-molecular interactions.

Thus far, our biophysical and imaging data suggest that cardiotoxin VII4 can target mitochondria to disrupt mitochondrial function in neurons, presumably by interacting with CL to form non-bilayer structures and thereby permeabilize mitochondrial membranes. To this end, we surmised that basic residues within cardiotoxin VII4 can interact with anionic phosphate head groups of CL in the OMM and IMM. To address this hypothesis, we performed molecular dynamics simulations to elucidate the molecular mechanism by which cardiotoxin VII4 interacts with a 1-palmitoyl-2-oleoyl-phosphatidylcholine (POPC) bilayer in the absence and presence of CL by using the Gromos53a6 force field [34]. Figure 10 shows the system setup of a typical molecular dynamics run, which entailed positioning the crystal structure of cardiotoxin VII4 over the POPC bilayer containing several molecules of cardiolipin (CL). After running the simulation trajectories of the system under constant NPT (N = moles, P = pressure, T = temperature) conditions for two simulation runs of 20 ns each (n = 2), we showed that cardiotoxin VII4 interacted with at least two CL phospholipids (Figure 11). 

We ensured first that the toxin itself remains stable in a solvent environment by performing a short equilibration simulation without a bilayer. This structure was then used for further simulations in a lipid-bilayer environment. Since the protein is seen to interact with the bilayer within 20 ns, we limited the production runs to 20 ns for both with and without CL.

Cardiotoxin VII4 rapidly bound with one CL molecule and subsequently interacted with a second CL molecule (Figure 11A). To determine the extent to which other phospholipids can be targeted by cardiotoxin VII4 (PDB: 1CDT), we simulated the interaction of cardiotoxin VII4 with an in silico lipid bilayer that consisted of POPC alone. In brief, time evolution analyses of each molecular dynamic run shows that cardiotoxin VII4 (shown by red line) failed to interact with the lipid bilayer that lacks CL (Figure 11B) as evident by an inability of cardiotoxin VII4 to reach the inter-atomic threshold of interaction with POPC molecules (dashed line), suggesting that cardiotoxin VII4 is electrostatically attracted to CL and not to PC (Figure 11). To further corroborate the specificity of cardiotoxins towards CL, we observed a similar result when running the same molecular dynamics experiments, but with crystal structures from other cardiotoxins of different cobra species, including cobra cardiotoxin CTX1 and cardiotoxin II from *Naja atra* (green and black lines representing 2CRT and 2CDX of Figure 11B). Our molecular dynamics data suggest that positively charged CVCs from various cobra species are attracted to phospholipids containing CL.

**Figure 12:**
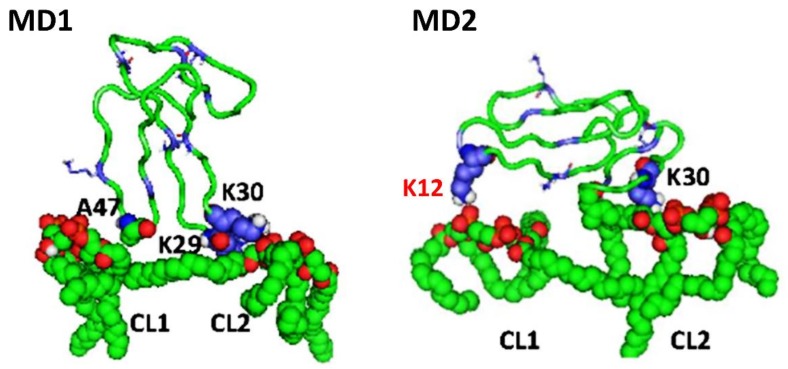
Molecular mechanisms by which cardiotoxin VII4 electrostatically interacts with CL in POPC bilayers. Molecular dynamics (MD) simulations showing that cardiotoxin VII4 has potential to bind up to two CL molecules by interacting with several lysine residues located in the N-terminal region of cardiotoxin VII4. The molecular models shown in this figure depicts K29 is unable to interact electrostatically with either phosphate or acyl groups of CL whereas K30, but not K12 (red), binds CL in both modes of interaction.

Next, in order to analyze the residues within cardiotoxin VII4 that interact with CL molecules of the simulated OMM, non-essential molecules were removed (H_2_O and POPC) to enhance the visibility of the atoms in CL molecules that interact with cardiotoxin VII4 (Figure 12). In brief, we observed that cardiotoxin VII4 binds to the phosphate groups of CL on the membrane surface by electrostatically associating with Lys 12, a residue that flanks loop 1, and with Lys 30, a residue located in the middle of loop 2 (Figure 12). Overall, our molecular dynamics data suggest that cardiotoxin VII4 is capable of interacting CL molecules via electrostatic and hydrogen bonding. 

### 2.9. Molecular Docking Studies Identified CL Binding Sites within Cardiotoxin VII4

To identify putative lipid-binding sites on the molecular surface of cardiotoxin VII4, we performed molecular docking simulations to analyze the interaction of cardiotoxin VII4 with either polar heads of CL and PC alone, which were generated by truncating the alkyl chains of phospholipids in silico or with the complete molecules of CL and PC. Given that alkyl chains of phospholipids are confined within the hydrophobic regions of the lipid bilayers, we surmised that specific charged groups IN CL and PC (Figures S1 and S2) and not the alkyl chains are not expected to interact with the cardiotoxin during the initial phase of long-range interactions between the cardiotoxin and the lipid bilayer. To this end, we initially performed molecular docking runs of cardiotoxin VII4 with just the polar heads of phospholipids (CL or PC). Secondly, we performed additional molecular docking runs between cardiotoxin VII4 and the complete molecule of CL or PC in order to simulate the ability of cardiotoxin to penetrate the hydrophobic core of a lipid bilayer as suggested by our biophysical data (Figure 9).

**Figure 13:**
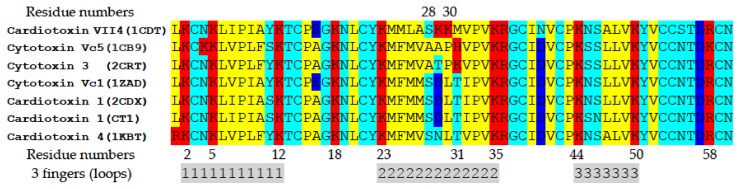
Amino acid sequence alignment of cobra venom cardiotoxins/cytotoxins, with resolved crystal structures, including cardiotoxin VII4 from *Naja mossambica mossambica*, 1CB9 from *Naja naja oxiana*, 2CRT from *Naja naja atra*, 1ZAD from *Naja naja oxiana*, and 2CDX from *Naja naja atra*. Basic and acidic residues are highlighted in red and blue, respectively. Polar and apolar residues are highlighted in light blue and yellow, respectively. The amino acid residues located within each of the three loops (I-III) are highlighted in gray below the amino acid sequences. The variable residues, Ser28 and Pro30, in loop 2, characteristic of S and P-type cytotoxins, respectively, are indicated by numbers 28 and 30 above the amino acid sequences.

Overall, our compiled molecular docking runs (polar head of CL or PC or the complete molecule of CL or PC) produced up to nine top ranked putative docked phospholipid-cardiotoxin structures that contained the lowest binding energies (interpreted as the maximum enthalpies released upon the binding of cardiotoxin with lipids). Overall, we observed that the interaction of cardiotoxin with each of the four lipid “ligands” involved ionic, ion–polar, and hydrogen interactions whereas the binding of the complete molecule of CL or PC with cardiotoxin predominantly involved van der Waals forces formed by the interactions of the alkyl chains of the phospholipids and hydrophobic surface areas of cardiotoxin (Tables S1 to S4). It is worth noting that ionic interactions between the cardiotoxin with each of the four ligands (phospholipid heads alone or the complete phospholipid) involved the interaction of the phosphate groups of CL or PC with most of the 12 basic amino acid residues of cardiotoxin, except for Lys2 and Lys30. Specifically, when cardiotoxin was docked with only the polar head of CL, we identified the following interacting basic amino acid residues in cardiotoxin VII4: K5, K12, K18, K35, R36, and K44. Secondly, when cardiotoxin was docked with the complete molecule of CL, we identified the following interacting amino acid residues in cardiotoxin VII4: K12, K18, K23, K35, R36, and R58. In addition, when cardiotoxin VII4 was docked with the polar head of PC, we identified the following interacting basic residues: K12, K18, K35, R36, and R58. Finally, the following interacting basic residues in cardiotoxin VII4 were observed to interact with the complete molecule of PC: K12, K18, K29, K35, K44, K50, and R36. However, it is worth noting that all basic residues in cardiotoxin VII4 that interacted with the polar heads of CL released more binding enthalpy energy per docked structure compared to cardiotoxin VII4 complexed with the polar head of PC. These data suggest that cardiotoxin VII4 binds more avidly to CL relative to PC (Tables S1 and S3). Interestingly, in all candidate docked structures, we identified four basic residues, K12, K18, K35 and R36, in cardiotoxins that were predominantly involved in binding to phospholipids. It is worth noting that these four lipid-binding residues are conserved among cardiotoxin across all species of cobra (Figure 13). Consistent with a previous study [19], our molecular docking data revealed that these four conserved basic amino acid residues, which are conserved in an S-type cytotoxin from *Naja oxiana* venom, formed ionic bonds by interacting with phosphate groups from PC and CL. These observations suggest that these four residues might be conserved CL-binding sites on the surface of S-types cardiotoxins/cytoxins. Furthermore, it should be noted that the enthalpy energy released when cardiotoxin VII4 docked to the complete structure of the phospholipids was higher relative to the enthalpy energy released when cardiotoxin VII4 docked to only the polar head groups (Tables S1 to S4). This data suggests that the alkyl chains from the lipids are critical for interacting with cardiotoxin at the lipid bilayer. Interestingly, we observed that a basic choline group in PC was unable to interact with the acidic residues of cardiotoxin (E16 and D57). In most docked structures, it is worth noting that this particular choline group was observed to be oriented away from the molecular surface of cardiotoxin, presumably to engage with other phospholipids of neighboring cardiotoxin bound to the membrane. 

Overall, while our molecular dynamics data reveal the molecular mechanisms by which cardiotoxin VII4 is electrostatically attracted to the surface of the OMM (Figure 10 and Figure 11), our molecular docking data identified CL-binding sites and revealed a molecular mechanism that explains how cardiotoxin VII4 can embed into mitochondrial lipid bilayers to permeabilize mitochondria.

## 3. Discussion

Membrane-active proteins from cobra venom, also known as cardiotoxins or cytotoxins, have been previously employed as valuable molecular tools to study the structure and functions of biological membranes [19,20,21,22,23,24,30,31,35]. Indeed, there is a wealth of experimental data that shows that cardiotoxins are membrane-remodeling proteins as they can bind to anionic phospholipids of cell membranes to alter the natural packing of lipids, leading to the formation of transmembrane pores and/or non-bilayer structures [21], and eventual cell death [20]. A study showed that cobra venom cardiotoxin targets mitochondria to induce mitochondrial dysfunction and ensuing apoptosis [36]. This observation poses the question of whether the cytotoxic action of cobra venom cardiotoxins is produced via its interaction with the cell membrane or via its interaction with the membrane of subcellular organelles—namely mitochondria. In our recent studies, we have shown that cytotoxins from *Naja oxiana* cobra venom specifically target CL in isolated mitochondria and in model membranes enriched with CL to induce the formation of non-bilayer structures within mitochondrial membranes, presumably in the IMM [19,30,36]. Intriguingly, at very low concentrations, cobra cytotoxins increased the activity of mitochondrial ATP-synthase whereas higher concentrations of cytotoxins completely abolished ATP-synthase activity, presumably by permeabilizing and consequently disintegrating mitochondrial membranes via the formation of toroidal pores [30,35]. 

In this study, we have shown that cardiotoxin VII4 from *Naja mossambica mossambica* cobra venom translocates to mitochondria (colocalization of Rhodamine-conjugated cardiotoxin with respiring mitochondria) in mouse cortical neurons and SH-SY5Y neuroblastoma cells to promote aberrant mitochondrial fragmentation/swelling (Figure 5), reduce mitochondrial respiration (Figure 6 and Figure 7) and ATP synthesis (Figure 2), and elicit neurodegeneration (Figure 1, Figure 2, Figure 3 and Figure 4). In neurons, the rapid translocation of cardiotoxin VII4 to mitochondria (<2 h.) coincides with a concomitant robust decrease in oxidative phosphorylation and of mitochondrial-derived ATP levels at 4 h post-treatment, early events that are upstream of neurodegeneration. We further showed that the ability of cardiotoxin VII4 to colocalize with mitochondria (Figure 3 and Figure 4) in live neurons/neuronal cells is associated with its ability to interact with CL in model membranes (Figure 8, Figure 9, Figure 10, Figure 11 and Figure 12, Table 1 and Table 2). Overall, our imaging and biophysical data support the notion that cobra cardiotoxin VII4 exhibit mito-toxic activity at high concentrations, presumably by interacting with CL to form non-bilayer structures (e.g., toroidal pores) to disrupt the mitochondrial membrane and structure. 

Like CTI and CTII from *Naja naja oxiana* cobra venom, we found that cardiotoxin VII4 preferentially binds to anionic phospholipids (CL), but not PC. Our data support the concept that highly basic three-fingered S and P-type cardiotoxins target the mitochondrion. Indeed, our erythrosine phosphorescence quenching data showed that cardiotoxin VII4 does not bind to pure PC membranes, but binds to PC membranes containing molar concentrations of CL as low as 3% to 5% (Table 1), a molar concentration of CL as found in the OMM [9]. The method of EPR of oriented multibilayer films, which we employed to investigate the effects of cardiotoxin VII4 on the phospholipid packing in pure PC membranes containing CL, showed that cardiotoxin VII4 does not affect phospholipid packing in pure PC membranes, presumably because cardiotoxin VII4 does bind to lipid membranes that consist of PC. However, we observed that cardiotoxin VII4 binds to PC membranes containing CL to decrease phospholipid mobility and induce the formation of non-bilayer structures (Table 2 and Figure 8). Most notably, the ability of cardiotoxin VII4 to induce non-bilayer structures was more pronounced in PC membranes containing 20 molar percent of CL than in the PC membranes containing 5 molar percent of CL (Figure 8 and Table 2). 

Our ^1^H-NMR spectroscopy data showed that cardiotoxin VII4 does not interact with pure PC liposomes, but interacts with CL to enhance the permeability of PC liposomes containing CL. Cardiotoxin VII4 also potentiates the formation of non-bilayer structures in CL containing liposomes as evident by the appearance of the high-field resonance peak for the outer N^+^(CH_3_)_3_ signal, which is more pronounced in liposomes containing 20 mol% CL relative to liposomes containing 5 mol% CL (Figure 9). This interesting observation suggests that PC and CL molecules contribute to the formation non-bilayer structures induced by the interaction of cardiotoxin VII4 with PC membranes containing CL.

**Figure 14:**
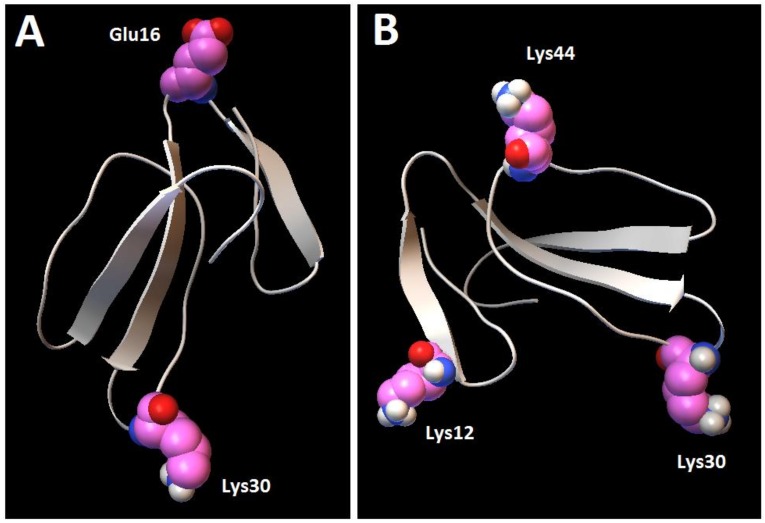
Interacting residues in cardiotoxin VII4 when oriented two different ways relative to the lipid bilayer. Ribbon diagrams of cardiotoxin VII4 showing that charged amino residues (ball and stick representations) are predicted to interact with the phosphate groups of CL (Lys12, Lys30, Lys44) and the choline group of PC (Glu16). **A**: ribbon diagram showing the interacting amino acid residues in cardiotoxin VII4 when it is oriented parallel to the membrane surface normal; **B**: ribbon diagram showing the interacting amino acid residues in cardiotoxin VII4 when it is oriented perpendicular to the membrane surface normal.

Importantly, our molecular dynamics data agree with our biophysical data in that cardiotoxin VII4 binds more avidly to CL relative to PC in lipid membranes of a similar phospholipid as that found in the OMM (PC + CL). Indeed, our molecular dynamics and biophysical data showed that cardiotoxin VII4 was unable to interact with PC lipid bilayers that lacked CL. In addition, our molecular dynamics data showed that cardiotoxin VII4 binds to CL molecules via two modes of interactions (Figure 12). The first mode of interaction (Figure 12, MD1) involves the binding of cardiotoxin VII4 to two CL molecules through electrostatic (via Lys 30 of the loop 2) and hydrogen bonds (via Ala47 of the loop 3), which ensures an orientation in which the long axis of the cardiotoxin is parallel to the membrane surface normal. Hence, should cardiotoxin VII4 embed into the lipid bilayer in this orientation, it will expose the acidic amino acid residue, Glu16, located on the opposite side of cardiotoxin VII4 (Figure 14A), presumably to interact with the basic choline groups of PC from neighboring regions of the lipid bilayer. Therefore, this mode of cardiotoxin–membrane interaction should facilitate intermembrane contacts, and may lead to the formation of inverted micelles, which contain a cardiotoxin at the core of a micelle, in a similar mechanism as described in previous studies [19,20,30]. When cardiotoxin is dissolved in solution, Glu16 cannot interact with choline groups of PC on the surface of the lipid bilayer due to the high positive force field on the surface of cardiotoxin. However, following the initial electrostatic interactions with CL on the OMM, it is likely that the solvent-exposed Glu 16 may facilitate the penetration of cardiotoxin into the lipid bilayer by interacting with the choline groups of PC from neighboring regions of the OMM. 

In the second mode of interaction (Figure 12, MD2), cardiotoxin VII4 binds to two CL molecules at the surface of the lipid bilayer via electrostatic forces that involve the interaction of two basic residues (Lys 30 and Lys12) in cardiotoxin with the phosphate groups of CL. Hence, in this mode, the long axis of cardiotoxin VII4 is oriented perpendicular to the membrane surface normal, thus exposing Lys44 to the cytosolic side of the OMM, which allows Lys44 to interact with the phosphate groups of CL of a neighboring lipid region (Figure 14B) in a similar manner as described in a previous study [19]. Interestingly, both basic amino acid residues located within loop 2 of cardiotoxin VII4 are not conserved among cobra venom cardiotoxins/cytotoxins (Figure 13), suggesting a unique mechanism by which the basic molecular surface of cardiotoxin VII4 can bind to the OMM. Interestingly, it is worth noting that cardiotoxin VII4 is the only known S-type cardiotoxin/cytotoxin that contains basic residues within loop 2. In contrast, other P-type cytotoxins (e.g., 1CD9 and 2CRT) contain positively charged amino acid residues (His31 or Lys31) within loop 2, which are distinct from VII4 (Figure 13). It is believed that basic residues and surrounding apolar amino acid residues with loop 2 facilitate the insertion of cytotoxins into lipid bilayers, whereas acidic and polar residues in loop 2 of most S-type cytotoxins lack lipid bilayer-penetrating activity [20]. However, it is conceivable that Lys30, which considerably extends from the molecular surface of cardiotoxin VII4 (Figure 14A), targets the phosphate groups of CL to ensure the insertion of cardiotoxin in an orientation that involves positioning the long axis of cardiotoxin VII4 parallel to the membrane surface normal. Hence, it is not likely that Lys29 directly interacts with a phosphate group of CL (Figure 12), but may facilitate the initial electrostatic interactions of cardiotoxin with the phosphate groups’ CL of the lipid bilayer prior to insertion into the OMM.

In another attempt to characterize the CL and PC biding sites on the molecular surface of cardiotoxin VII4, we performed molecular docking studies. Hence, by cosidering the ability of cardiotoxin to interact with the phospholipid head groups, our docking data provides insight on how cardiotoxin interacts with the phosphate groups of CL prior to embedding into the hydrophobic regions of the simulated IMM. In brief, our molecular docking data show that four basic residues (Lys12, Lys18, Lys35, and His36) formed ionic bonds with the phosphate groups of CL (Table S1) whereas three basic residues (Lys12, Lys18, and His36) formed ionic bonds with a phosphate group of PC (Table S3). These results differ from our molecular dynamics data, which show that VII4 does not bind to PC, but preferentially binds to CL via long-range ionic interactions between the phosphate groups of CL and the −N^+^H_3_ group of Lys12 and Lys30. This apparent discrepancy between our molecular docking and molecular dynamics data is likely due to the fact that the alkyl chains of lipids, whose molecular motion is restricted within hydrophobic regions of the lipid bilayers, does not allow the docking of a lipid polar head with the cardiotoxin, and likely resembles interactions between the protein and a membrane surface. Therefore, our molecular docking data likely informs on the interaction of cardiotoxins with the alkyl chains of PC and CL once it has successfully penetrated the hydrophobic core of the lipid bilayer (OMM or IMM). Nevertheless, the most stable docked structures of cardiotoxin VII4 with the CL polar head involved ionic interactions between Lys 12 and the phosphate group (Table S1), which is consistent with our MD simulations data (Figure 12, MD2). On the other hand, Lys30 of cardiotoxin VII4 was not identified as an interactive amino acid residue in the docked structures of cardiotoxin with the polar heads of CL or PC, presumably because the −N^+^H_3_ group of Lys30 is far from the surface of cardiotoxin. It is conceivable that the small positively charged surface area contributed by the –N^+^H_3_ group of Lys30 may interact with the choline groups of PC to enable the cardiotoxin to penetrate the membrane surface. 

It should be noted that the binding enthalpy energy released for all putative docked cardiotoxin VII4-CL polar head complexes was observed to be relatively higher than the enthalpy energy released when cardiotoxin VII4 docked to the polar heads of PC (Tables S1 and S3). These data support the notion that cardiotoxin VII4 preferentially binds to CL on the surface of lipid bilayers of the OMM. Additionally, the binding enthalpy energy released for all putative docked structures for cardiotoxin VII4 complexed to the complete structure of CL was observed to be lower than that for the docked structures of cardiotoxin VII4 complexed to the complete PC molecule (Tables S2 and S4). These data suggest that PC molecules bind more strongly to cardiotoxin VII4 compared to CL when cardiotoxin VII4 embeds into the membrane hydrophobic area of lipid membranes. In addition, our in silico data suggest that hydrophobic interactions generated by the two long alkyl chains of PC play a major role in enabling the cardiotoxin to penetrate the lipid bilayer. In addition, the four alkyl chains from CL may facilitate the formation of non-bilayer structures known to permeabilize CL-containing lipid bilayers.

An inherent limitation of our docking simulations is the lack of flexibility of the protein, which is held fixed in most docking algorithms. While MD simulations can in principle be used to evaluate molecular flexibility, for large proteins, the conformational changes may occur over several dozens of nanoseconds. A way to overcome this limitation is to generate multiple structures (conformations) of the protein using various softwares (e.g., Confab, OMEGA, and PC Spartan Pro). While it would be preferable to probe the conformational profile of ligands in the enzyme active site to significantly improve the accuracy of docking studies [37,38], we employed the highest level of flexibility allowed by AutoDock by using the exhaustiveness level 16, and performed three docking simulations for each ligand for statistical purposes. Hence, running docking studies using the aforementioned paramaters can add more rigor and accuracy to our docking studies and can partially account for the flexibility and conformational changes that lipids can undergo (~dozens of nanoseconds).

Overall, based on our compiled and in silico data, we suggest the following molecular mechanism by which cardiotoxin VII4 targets mitochondria to disrupt mitochondrial function. First, our collective data suggest that cardiotoxin VII4 penetrates the plasma membranes via a similar mechanism previously described for cytotoxins derived from *Naja oxiana* cobra venom [19]. In brief, cardiotoxin VII4 binds to anionic phospholipids on the plasma membrane of a neuron to dehydrate the lipid surface, alter the asymmetry of the lipid bilayer to form immobile non-bilayer structures, and subsequently inserts into hydrophobic area of the membrane with the long axis oriented parallel to the membrane surface normal. However, unlike cytotoxins (CTI and CTII), an acidic residue, Gly16, located on the opposite side of cardiotoxin VII4 may attract the basic choline groups of PC from a neighboring membrane (e.g., a membrane of the synaptic juncture), presumably to facilitate the formation of non-bilayer structures, including inverted micelles that may contain cardiotoxin VII4 within the center of the inverted micelle. The inverted micelle then releases cardiotoxin VII4 to the cytosolic compartment of neurons to target the OMM, and subsequently interact with OMM-IMM contact sites enriched in CL. Therefore, the biochemical environment of the OMM, which is enriched with inverted CL, may facilitate the translocation of cardiotoxin VII4 into a junction between OMM and IMM, where it generates inverted micelles in the intermembrane contact sites via interactions of the conserved basic residues, Lys12, Lys30, and Lys44 (Figure 14), with CL molecules of the OMM and IMM. An inverted micelle containing cardiotoxin VII4 within a contact site between the OMM and IMM may thereby release the cardiotoxin into the intermembrane space or into the mitochondrial matrix, where cardiotoxin may bind to the mitochondrial cristae to disrupt the IMM, leading to mitochondrial fission/swelling of the mitochondrial complexes, leading to a reduction in mitochondrial oxidative phosphorylation and ATP production. 

It is worth noting that the membrane-active properties of cardiotoxin VII4, as supported by our collective biophysical data, are highly similar to the membrane-active properties of cytotoxin CTII from *Naja oxiana* cobra venom [19]. Therefore, it is conceivable that cardiotoxin VII4, similarly to cytotoxin CTII, may affect mitochondrial structure and function in a concentration-dependent manner. At low concentrations, cytotoxin CTII can increase ATP-synthase activity to increase the steady-state level of ATP [30,31], whereas high concentrations of cytotoxin are detrimental to the mitochondrial structure by the formation of oligomers. Future directions warrant investigating the effects of very low concentrations of cardiotoxin VII4 on ATP-synthase activity and mitochondrial structure in intact mitochondria as previously described [30]. Should cardiotoxin VII4 prove to increase mitochondrial ATP-synthase activity at very low concentrations while maintaining an intact mitochondrial structure, cardiotoxin VII4 may represent a promising pharmacological avenue for improving the mitochondrial bioenergetics in neurons, which can be employed for the treatment of patients with the age-depended neurodegeneration conditions. 

## 4. Conclusions

In conclusion, our compiled data suggest that highly basic three-fingered cobra venom cardiotoxins exhibit neurotoxicity by inducing mitochondrial pathology in a similar manner as amylodogenic proteins (α-synuclein, β amyloid, Tau) by targeting CL. Although cobra venom cardiotoxins do not contain an amyloid-like domain, these non-enzymatic proteins possess common biophysico-chemical properties similar to amyloid-like proteins (highly basic charge, interspersed hydrophobic regions at the core structure, lipid penetrating finger domains) that enable them to target CL and subsequently penetrate the OMM to form toroid-like structures. Our data warrants future studies to further characterize the “mito-toxic” potential in other highly basic snake venom cardiotoxins, and further refine our conceptual model by which cardiotoxins promote neurotoxicity.

## 5. Materials and Methods

### 5.1. Molecular Reagents

Egg yolk L-α-phosphatidylcholine (PC), cardiolipin (CL) from *Escherichia coli*, the EPR spin-labeled probes 5-doxylstearic acid (5-DSA), and potassium ferricyanide were all purchased from Sigma Chemical Co. (St. Louis, MO, USA). All phospholipids were further purified on silica columns. Erythrosine (Sigma-Aldrich, St. Louis, UK) was used as a phosphorescent probe while ferrocene (Sigma Chemical Co., St. Louis, MO, USA) was used as a quencher of erythrosine phosphorescence. 

Pure cardiotoxin from *Naja naja mossambica* was purchased from Sigma-Aldrich (St.Louis, MO, USA) (C9759). The NHS-Rhodamine conjugate alone and a NHS-Rhodamine Antibody Labeling kit (53031) were purchased from Thermo Scientific (Waltham, MA, USA). A CytoTox-ONE^TM^ Homogenous Membrane Integrity Assay (G7890) was purchased from Promega (Madison, WI, USA). All other reagents were purchased from Life Technologies (Grand Island, NY, USA), including MitoTracker Green, Dulbecco’s Modified Eagle’s Medium (DMEM), Neurobasal Medium, B-27 Supplement, Glutamax, poly-L-lysine, fetal bovine serum, and sodium pyruvate. D-glucose, glutamic acid, and D-galactose were obtained from Thermo Scientific.

### 5.2. Handling of Timed-Pregnant Mice and Ethics Statement

All experiments involving mice were done in accordance to the Animal Research: Reporting of In Vivo Experiments (ARRIVE) guidelines and in adherence to the Guide for the Care and Use of Laboratory Animals (National Institutes of Health). All animal procedures used to minimize distress were specifically approved for research by the Institutional Animal Care and Use Committee of the University of Nevada, Reno (Protocol # 00572, approved on January 18, 2019 for three years, Animal Welfare Assurance Number: Issued by OLAW: A3500-01). Specific pathogen free (SPF) 14 day timed pregnant female C57/BL6 mice were ordered from Charles River (Wilmington, MA) and housed for two to three days upon arrival at the specific-pathogen free-approved Center for Molecular Medicine Animal Facility (UNR).

### 5.3. Cell Culture

Primary cortical neurons were prepared by extracting the cortices of C57BL/6J fetal mice as previously described [32,39]. In brief, primary cortical neurons were plated on poly-L-lysine pre-coated plates at a cell density of 200,000 cells per well in 24 well plates, in 96 well plates for the analyses of cell survival, and in 4 well chambered coverglasses (Nunc (Thermo Scientific)) for performing confocal microscopy-mediated analysis of the colocalization of NHS-Rhodamine labeled-cardiotoxin with mitochondria labeled with MitoTracker Green. The primary cortical neurons were initially plated in Neurobasal plating media, (94.8% neurobasal medium, 2% B-27 supplement, 0.2% glutamic acid, 2% fetal bovine serum, and 2 mM Glutamax). Three days later, two thirds of the media was removed and replaced with neurobasal maintenance media (97% neurobasal medium, 2% B-27 supplement, and 2.0 mM Glutamax). SH-SY5Y neuroblastoma cells (ATCC, Manassas, VA, USA) from pre-existing passages were purchased from the American Tissue Culture Collection and maintained up to 27 passages. SH-SY5Y neuroblastoma were maintained in complete DMEM medium (DMEM 90%, 10% fetal bovine serum, 2 mM Glutamax and supplemented with 5 mM HEPES) per the cell culture instructions published by American Tissue Culture Collection (ATCC).

### 5.4. LDH Assay

To analyze for the effects of snake venom cardiotoxin VII4 on cell viability, the CytoTox-ONETM Homogenous Membrane Integrity Assay kit (G7890) (Madison, WI, USA) was utilized per the manufacturer’s instructions. After primary cortical neurons or neuronal cells (SH-SY5Y cells) reached up to 85% confluency on black-sided Corning 96-well plates (Fisher Scientific, Waltham, MA, USA), the cells were exposed to increasing concentrations of VII4 (0.5 mM–2.0 μM, and 2.5 μM) in primary neurons or SH-SY5Y neuroblastoma cells (1.0 mM–10.0 μM ) for 24 h prior to measuring the release of lactate dehydrogenase (LD) as measured by fluorescence spectroscopy using an excitation wavelength of 560 nm and emission wavelength of 590 nm on a Molecular Devices SpectraMax M3 plate reader.

### 5.5. ATP Assays

SH-SY5Y neuroblastoma cells were plated at a density of 100,000 cells per well on a black-sided, clear bottom 96-well plate. Each condition was set up in triplicate wells. After 24 h of culture, the initial complete DMEM media was removed and the cells were gently washed with DMEM containing 10 mM galactose, 2 mM sodium pyruvate, 2 mM glutamine, and 10% FBS (DMEM-Gal). The cells were then left in DMEM-Gal media for another 24 h to simulate enhanced mitochondrial respiration and thereby increase ATP derived from mitochondria [40]. For some wells, cardiotoxin VII4 was added to designated wells with cells for 4 h before the assay analysis. Four hours following exposure with DMEM-Gal media and VII4, another set of designated wells was replaced with DMEM-Gal media containing 1 μM oligomycin as the final treatment. Oligomycin is an ATP-synthase inhibitor, and is a molecular tool used to inhibit ATP synthase-dependent ATP production and thereby calculate the amount of ATP derived from mitochondria. For each ATP assay, the final experimental conditions were: 1. DMEM-Gal only; 2. DMEM-Gal + VII4; 3. DMEM-Gal + Oligomycin; and 4. DMEM-Gal + Oligomycin + VII4. ATP standards were prepared in 1:10 serial dilutions in DMEM-Gal media to generate a standard curve (10 μM, 1 μM, 0.1 μM, 0.01 μM, and 0.001 mM). 100 μL of Cell Titer-Glo^®^ Reagent (Promega, Madison, WI, USA) was added to all well conditions, the ATP standards, and the plate blank wells. The plate was then wrapped in aluminum foil and incubated at room temperature (22 °C) for 2 min on an orbital shaker. Lastly, the plate was read by employing a Molecular Devices SpectraMax M3 (Molecular Devices, San Jose, CA, USA) plate reader on the luminometry setting for one reading and normalized to the protein concentration as previously described [40,41].

### 5.6. Chemical Labeling of Cardiotoxin with Rhodamine and Confocal Microscopy

In order to follow the ability of VII4 to translocate to mitochondria by employing confocal microscopy, rhodamine was conjugated to the cardiotoxin by following the manufacturer’s protocol for the NHS-Rhodamine Antibody Labeling Kit (Pierce, Rockford, IL, USA). In brief, 1 mg of solid VII4 was used for each conjugation to make a final concentration of a 295.7 μM stock solution. The chemical conjugation of NHS-Rhodamine with cardiotoxin VII4 was successfully corroborated by using the following formula:

Moles NHS labeled Rhodamine per mole of protein = (Amax of labeled protein fluor × protein concentration) × (dilution factor)

To follow the uptake of VII4 and colocalization with mitochondria in cells by confocal microscopy, the mitochondria in primary cortical neurons and SH-SY5Y neuroblastoma were stained with a 150 nM solution of MitoTracker Green FM (Thermo Fisher Scientific, Waltham, MA, USA) in Neurobasal media or with complete DMEM media, respectively. Up to 10–15 immunofluorescent TIFF images per condition were captured by employing an Olympus Fluoview 1000 confocal microscope (Olympus) at a 60 × magnification, 4.0 digital zoom factor, sequential capture setting, and by applying the Kollman’s filter. The percentage of mitochondria (green channel) that colocalize (yellow) with Rhodamine labeled-VII4 (red channel) was analyzed by using NIH Image J version 1.44 and by using similar fluorescent imaging-based quantifications previously described for neurons [32].

### 5.7. Bioenergetic Analysis: Mitochondrial Oxygen Consumption Assays

In order to measure the effects of cardiotoxin on mitochondrial respiration in neuroblastoma cells as a proxy of oxidative phosphorylation activity, a mitochondrial stress assay was performed by using an XF24e Extracellular Flux Analyzer (Seahorse Biosciences, Agilent Technologies) as previously described [42], but with the following minor modifications. In brief, this assay was done by first plating SH-SY5Y cells at a density of 70,000 per well in complete medium (DMEM with 10% FBS) on a XF24 well cell culture microplate (Agilent Technologies). Cells were then exposed to cardiotoxin at the LD_50_ (1.3 µM) in quadruplicate wells. Wells, B4 and C3, of the XF24 cell culture microplate were assigned as blanks (no cells) in order to assess background oxygen consumption rates. A calibration plate was also prepared by adding 1 mL of calibrant fluid (SeaHorse Bioscience, North Billerica, MA, USA) into the plate wells one day prior to performing the mitochondrial stress assay. On the day of the assay, the cells were washed with XF Media (XF Media (Seahorse), glucose (4.5 g/1 L), 1 mM sodium pyruvate, 1 mM glutamine). Oxygen consumption rates (OCRs) were analyzed at baseline and following the sequential addition of 1 μm oligomycin, 300 nm FCCP, and 1 μm rotenone/antimycin A to determine the ATP-linked OCRs, maximal OCRs, and mitochondrially-derived OCRs, respectively, as published previously [42]. The final concentrations of oligomycin, FCCP, and rotenone/ antimycin-A were 1 μM each and were prepared in the XF Assay media in order to assess for ATP-dependent, maximal, and mitochondrial-specific oxygen consumption rates, respectively. The machine was programmed to expose the cells to cardiotoxin VII4 for 22 cycles (3 h) to measure the oxygen consumption rate (OCR). At the end of each assay, the OCRs and ECARs were normalized to the protein concentration (μg/mL) for their respective well.

### 5.8. Erythrosine Phosphorescence Quenching Studies

Unilamellar liposomes for erythrosine phosphorescence quenching assays were prepared as previously described [21]. In brief, liposomes were incubated with 5 × 10^−6^ M erythrosine for 20 h, followed by incubation with 10^−5^ M ferrocene for 2 h at 4 °C. To remove unbound probes, the liposomes were chromatographed on a Sephadex G 50 column (1.8 × 50 cm) as previously published [21]. For the liposomes used for phosphorescence assays, oxygen was enzymatically removed by glucose oxidase treatment. The analyzed liposome samples were contained in a square (10 mm) quartz chamber that was incubated at 18 °C. The erythrosine phosphorescence quenching by ferrocene in the presence or absence of defined cardiotoxin concentrations was detected with a filter specific for a wavelength greater than 700 nm following excitation with a pulsed laser apparatus (Chernogolovka, Russia). The lifetime of the excited state of erythrosine was estimated as the time dependence of the attenuation of the probe glow using semi-logarithmic coordinates. Samples for each data point were made and recorded in triplicate. The error of the erythrosine excited state lifetime was determined to be less than 5%. 

### 5.9. EPR Studies

For EPR studies, oriented multibilayer films were prepared as previously described [21]. Briefly, 50 μL phospholipid suspension in buffer (10 mM Tris-HCl, pH 7.5, 0.1 M NaCl, 1 mM EDTA) was squeezed between two glass plates. The phospholipid concentrations in suspensions were prepared at 5 mM and they were made of either pure PC or PC + CL in molar ratios 9.5 and 0.5 or 8 and 2, respectively. Phospholipid suspensions contained a 10^−5^ M 5-DSA spin probe and defined concentrations of cardiotoxin. EPR spectra of 5-DSA in multibilayer films were recorded with a Varian E-4 spectrometer (Palo Alto, CA USA) at modulation amplitudes not exceeding 2 × 10^−4^ T and with a resonator input power not exceeding 20 mW at 18 °C. The analysis of the EPR spectra was done in terms of the *B*/*C* ratio [21] and the *S* parameter [43]. *B* is defined as the intensity of the low-field component while *C* is defined as the intensity of the central component of EPR spectra taken with respect to the magnetic field perpendicular to the bilayer normal. The formula used to calculate the *S* parameter was previously published [43]. Each sample was prepared and analyzed by EPR in three technical replicates for each of three independent samples. The means and standard deviations of these measurements were used as experimental data points. The variation between the triplicates was observed to be less than 5%.

### 5.10. ^1^H-NMR Studies

Unilamellar liposomes for ^1^H-NMR studies were prepared by drying phospholipids in chloroform via the continuous exposure of the sample to helium in vacuum for 1.5 h in order to form a lipid film. The film was then hydrated in a ^2^H_2_O buffer containing 10 mM Tris-HCl, pH 7.4, and 0.5 mM EDTA. The lipid suspension was sonicated for 15 min in helium media at 4 °C by employing an ultrasonic disperser, USDN-1 (St. Petersburg, Russia), at a frequency of 22 kHz. Unilamellar liposomes were centrifuged at 200 × *g* for 60 min to remove heavy phospholipid aggregates and then incubated in helium atmosphere for 15 h at 10 °C. Unilamellar liposomes were made of either pure PC or PC + CL in molar ratios of 9.5 and 0.5 or 8 and 2, respectively. The total concentration of phospholipids in unilamellar liposomes was 1.4 × 10^−2^ M. Cardiotoxin VII4 dissolved in a ^2^H_2_O-containing buffer was added directly into unilamellar liposomes at concentration 2.1 × 10^−4^ M. ^1^H-NMR spectra from unilamellar liposomes were recorded at 18 °C at an operating frequency of 200 MHz. The width of the 90° pulse was 8.7 μs, the relaxation delay was 50 μs, and the acquisition time for the free induction signal was 1 s. To split ^1^H NMR signals from the N^+^(CH_3_)_3_ groups of PC in the outer and inner leaflets of unilamellar liposomes, 10 μL of shift reagent (saturated K_3_Fe(CN)_6_ solution in ^2^H_2_O buffer) was added per 1 mL of liposomes. The measurements of the integral intensity of ^1^H NMR signals from the N^+^(CH_3_)_3_ groups of PC were done in triplicate readings. The variation between the triplicates was observed to be less than 6%.

### 5.11. Molecular Dynamics

A homology model based on the solution NMR structure of cardiotoxin was built for cardiotoxin *Naja mossambica mossambica* (PDB ID: 1CDT). In brief, the homology model was energy minimized due to the variability of NMR structures as previously published [19]. The higher structural stability was determined by QMEAN scores, a scoring function for protein structures based on MD simulations and other protein models [42,43]. The processed protein template (cardiotoxin VII4) was then energy minimized using GROMACS version 4.5.1 with the GROMOS 53a6 force field parameters for proteins and lipids. The protein was then put into a system with a lipid bilayer with and without CL molecules and solvated.

Two simulations trajectories of 20 nanoseconds (ns) each were performed for both the cardiotoxin VII4 systems. The system consisted of the protein placed at a distance of 20 Å from a lipid bilayer made up of 128 molecules of 1-palmitoyl-2-oleoyl-phosphatidyl-choline (POPC) and three CL (CDL) molecules embedded in the bilayer, simulating the OMM. The cardiotoxin was placed about 20 Å away from the surface of the lipid bilayer, and the whole system was solvated with explicit SPC water molecules. Each simulation system was first energy minimized with 1000 steps of the steepest descent, followed by a short equilibration run of 200 picoseconds. During the equilibration, the protein backbone atoms were fixed and water and lipid molecules were allowed to relax. This was followed by 20 ns of the production run using GROMACS version 4.5.1 with GROMOS 53a6 force field parameters for the protein and lipids. The simulations were performed under constant NPT conditions, with an integration time step of 2 femtoseconds. The LINCS [44] algorithm was used to constrain all bond lengths and the Particle Mesh Ewald method [45] was used to compute the electrostatic interactions. The temperature was kept constant at T = 310 K by coupling to an external temperature bath with a coupling constant of 0.1 picosecond and a pressure coupling was employed to maintain a constant pressure of 1 bar. The force-field parameters for CDL was generated using PRODRG [46]. In addition, two control simulations of 20 ns each were also performed for the cardiotoxin VII4 system with a bilayer consisting of only POPC lipids. The simulation protocols for these simulations were the same as those for toxin simulations with POPC and CDL molecules.

### 5.12. Molecular Docking

In order to identify potential phospholipid binding sites in cardiotoxin VII4, the phospholipid head groups of CL and PC or the complete molecules of PC and CL (head groups and alkyl chains) were docked with the solution NMR structures of cardiotoxin VII4 by using the AutoDockVina Version 4.2 program using a similar methodology as previously published [47]. In brief, the PDB coordinates of CL were extracted from the bovine heart oxidoreductase crystal structure bound to CL (PDB ID# 1V54). The PDB coordinates of phosphatidylcholine (PC) were extracted from the structure of PITP complexed to DOPC (PDB ID# 1T27). The lipids were further edited to remove the alkyl chains using Avogadro as previously published [48], and the overall charges were checked and energy minimized using AutoDock. The phospholipid “ligands”, which consisted of the phospholipid head groups and the complete molecules of PC and CL, contained rotatable bonds whereas the cytotoxins were kept as rigid molecules for each run. The overall molecular surface of cardiotoxin VII4 was considered for these docking studies (blind docking). A grid box was set up with the following dimensions: A center of x = 13.277; center of y = 25.025; center of z = –0.456; length of x = 36 Å; length of y = 46 Å; length of z = 36 Å. The setting for exhaustiveness was set up as 16, which gave us consistent results in at least three sets of docking for each ligand and cardiotoxin VII4 pair in this study. It is important to note that the grid box was large enough to do a “blind” docking by encompassing the entire surface of the cytotoxin VII4 and a phospholipid “ligand” for each molecular docking simulation. Following each Autodock run, the best nine docked conformations were analyzed for ionic, ion–polar, and hydrogen bond interactions between phospholipid polar head groups and charged and polar amino acids of cardiotoxin VII4 by using Python Molecular Viewer (MGL Tools, The Scripps Research Institute).

### 5.13. Statistics

Unless indicated otherwise, most results are expressed as mean ± S.E.M. from three independent experiments or a representative experiment. Data was analyzed by Student’s t test (two-tailed) for single comparisons. Multiple group comparisons were done by performing one-way ANOVA followed by Bonferroni-corrected Tukey’s test. *P* values less than 0.05 were considered statistically significant.

## Figures and Tables

**Table 1:** Quantification of the erythrosine phosphorescence quenching by ferrocene in response to increasing molar percentage of cardiolipin (CL) in phosphatidylcholine (PC) liposomes that were treated with various concentrations of cardiotoxin VII4 (*Naja mossambicamossambica* cobra cardiotoxin).

Lifetime of Erythrosine Phosphorescence (Ƭn/Ƭo)												
0.0025 molar ratio	1.00 (0.019)	1.01 (0.018)	1.01 (0.019)	1.04 (0.018)	1.06 (0.021)	1.07 (0.020)	1.06 (0.022)	1.06 (0.019)	1.07 (0.018)	1.08 (0.021)	1.10 (0.020)	1.10 (0.024)
0.005 molar ratio	1.00 (0.020)	1.05 (0.022)	1.08 (0.021)	1.11 (0.028)	1.13 (0.019)	1.16 (0.022)	1.18 (0.026)	1.19 (0.018)	1.20 (0.031)	1.21 (0.025)	1.22 (0.029)	1.22 (0.019)
0.01 molar ratio	1.00 (0.020)	1.07 (0.024)	1.10 (0.025)	1.12 (0.018)	1.15 (0.021)	1.18 (0.023)	1.21 (0.027)	1.23 (0.025)	1.23 (0.022)	1.26 (0.020)	1.28 (0.021)	1.29 (0.019)
0.02 molar ratio	1.00 (0.018)	1.09 (0.021)	1.12 (0.020)	1.15 (0.019)	1.18 (0.030)	1.21 (0.035)	1.24 (0.028)	1.25 (0.021)	1.26 (0.020)	1.28 (0.028)	1.30 (0.026)	1.32 (0.038)
Molar percentage of CL in PC liposomes												
VII4/Lipid	0%	1%	2%	3%	4%	5%	6%	7%	8%	10%	15%	20%

Ƭn and Ƭo denote the lifetimes of erythrosine phosphorescence in the presence and absence of cardiotoxin, respectively, as indicated by blue numbers. The data are the compiled means from three independent erythrosine phosphorescence experiments (standard deviations are shown in parenthesis). Cardiotoxin VII4/lipid molar ratios are shown in a column with black numbers.

**Table 2:** The values of the *B*/*C* ratio and the *S* parameters were calculated from the EPR spectra of 5-DSA in oriented multibilayer lipid films treated with increasing concentrations of cardiotoxin VII4.

Cardiotoxin VII4/Lipid Molar Ratio	0% CL in PC Membranes		5% CL in PC Membranes		20% CL in PC Membranes	
	(B/C)_i_/(B/C)_o_	S_i_/S_o_	(B/C)_i_/(B/C)_o_	S_i_/S_o_	(B/C)_i_/(B/C)_o_	S_i_/S_o_
0.000	1.00 (0.018)	1.00 (0.019)	1.00 (0.016)	1.00 (0.018)	1.00 (0.019)	1.00 (0.021)
0.005	1.00 (0.021)	1.00 (0.020)	0.90 (0.013)	1.14 (0.020)	0.80 (0.012)	1.19 (0.025)
0.010	1.00 (0.016)	1.00 (0.018)	0.75 (0.015)	1.20 (0.022)	0.61 (0.012)	1.26 (0.026)
0.020	1.00 (0.018)	1.00 (0.022)	0.60 (0.012)	1.25 (0.024)	0.47 (0.010)	1.32 (0.028)
0.030	1.00 (0.019)	1.00 (0.023)	0.50 (0.010)	1.29 (0.029)	0.44 (0.008)	1.36 (0.033)
0.040	1.00 (0.020)	1.00 (0.018)	0.50 (0.011)	1.31 (0.030)	0.44 (0.009)	1.37 (0.032)

The data for the *B*/*C* ratios and the *S* parameters represent the means compiled from three independent experiments. The standard deviation values are given in parenthesis. Values for (*B/C*)_0_ and *S*_0_ represent experimental conditions in which multibilayer lipid films do not contain cardiotoxin VII4 whereas the values for (*B*/*C*)_i_ and *S*_i_ represent multibilayer lipid films treated at the indicated cardiotoxin VII4 concentrations.

## Supplementary Materials

The following are available online at https://www.mdpi.com/2072-6651/11/3/152/s1, Figure S1: The marking of the charged and polar groups in the CL polar head, Figure S2: The marking of the charged and polar groups in the PC polar head, Table S1: Summary of the charged and polar groups of amino acid residues (a.a.r.) in the VII4 binding sites that interact with polar head groups of truncated CL, Table S2: Summary of the charged and polar groups of amino acid residues (a.a.r.) in the VII4 binding sites that interact with polar head groups of complete CL molecule, Table S3: Summary of the charged and polar groups of amino acid residues (a.a.r.) in the VII4 binding sites that interact with polar head groups of truncated PC, Table S4: Summary of the charged and polar groups of amino acid residues (a.a.r.) in the VII4 binding sites that interact with polar head groups of complete PC.
