# Supplementary material for: Naja mossambica mossambica Cobra Cardiotoxin Targets Mitochondria to Disrupt Mitochondrial Membrane Structure and Function
Source: Toxins (Basel). 2019 Mar 8;11(3):152. doi: 10.3390/toxins11030152 (PMC6468758; doi:10.3390/toxins11030152)
Supplement: Supplementary file 1 [file toxins-11-00152-s001.pdf]

Supplementary Materials: Naja mossambica mossambica Cobra Cardiotoxin Targets Mitochondria to Disrupt Mitochondrial Membrane Structure and Function

Boris Zhang, Feng Li, Zhengyao Chen, Indira H. Shrivastava, Edward S. Gasanoff and Ruben K. Dagda

Table S1. Summary of the charged and polar groups of amino acid residues (a.a.r.) in the VII4 binding sites that interact with polar head groups of truncated CL. Hypothetical binding sites in VII4 that bind to the charged and polar groups of CL as determined by AutoDock modeling. Designation of PO^{4-} groups and carbon atoms in $CO^{\delta-}$, $CO^{\delta-}C$ and $C = O^{\delta-}$ in CL polar head is shown in Figure S1. Pb in $NH_{pb}^{\delta+}$ denotes a peptide bond.

Site number	CL polar group	VII4 a.a.r. polar group	Bond type
	A PO_4^-	$K12(N^+H_3)$ $C38 (NH_{pb}^{\delta+})$	Ionic, Ion-polar
Binding site 1	B PO_4^-	$K12(N^+H_3)$	Ionic
Affinity: −4.2 kcal/mol	1 $CO^{\delta-}C$	$K35(N^+H_3)$ $Y22(OH^{\delta+})$	Ion-polar, hydrogen
	2 $C = O^{\delta-}$	$K35(N^+H_3)$	Ion-polar
	A PO_4^-	$Y51(OH^{\delta+})$	Ion-polar
	B PO_4^-	$K35(NH_3^+)$ $Y22(OH^{\delta+})$	Ionic, Ion-polar
Binding site 2	1 $C = O^{\delta-}$	$K44(NH_{pb}^{\delta+})$	hydrogen
Affinity: −4.1 kcal/mol	$CO^{\delta-}$	$K35(N^+H_3)$	Ion-polar
	3 $CO^{\delta-}C$	$K18(N^+H_3)$	Ion-polar
	3 $C = O^{\delta-}$	$K18(N^+H_3)$	Ion-polar
	4 $C = O^{\delta-}$	$K12(N^+H_3)$ $C38(NH_{pb}^{\delta+})$	Ion-polar, hydrogen
Binding site 3 Affinity: −4.0 kcal/mol	A PO_4^-	$K35(N^+H_3)$	Ionic
	B PO_4^-	$C38(NH_{pb}^{\delta+})$ $K12(N^+H_3)$	Ion-polar, ionic
	$CO^{\delta-}$	$K35(N^+H_3)$ $Y22(OH^{\delta+})$	Ion-polar, hydrogen
	2 $C = O^{\delta-}$	$N40(NH_2^{\delta+})$	hydrogen
	3 $C = O^{\delta-}$	$K12(N^+H_3)$	Ion-polar
	B PO_4^-	$K35(N^+H_3)$	Ionic
Binding site 4	2 $C = O^{\delta-}$	$K5(N^+H_3)$	Ion-polar
Affinity: −3.7 kcal/mol	3 $C = O^{\delta-}$	$K12(N^+H_3)$	Ion-polar
	4 $C = O^{\delta-}$	$L6(NH_{pb}^{\delta+})$	Hydrogen
	A PO_4^-	$K18(N^+H_3)$	Ionic
Binding site 5	B PO_4^-	$K12(N^+H_3)$	Ionic
Affinity: −3.7 kcal/mol	2 $CO^{\delta-}C$	$K12(N^+H_3)$	Ion-polar
	3 $CO^{\delta-}C$	$L6(NH_{pb}^{\delta+})$	Hydrogen
Binding site 6 Affinity: −3.7 kcal/mol	A PO_4^-	$K35(N^+H_3)$ $Y22(OH^{\delta+})$	Ionic, Ion-polar
	B PO_4^-	$Y51(OH^{\delta+})$	Ion-polar
	1 $C = O^{\delta-}$	$N40(NH_2^{\delta+})$ $K18(N^+H_3)$	Hydrogen, Ion-polar,
	2 $C = O^{\delta-}$	$K18(N^+H_3)$	Ion-polar
	$CO^{\delta-}$	$Y51(OH^{\delta+})$	Hydrogen
	3 $C = O^{\delta-}$	$S46(OH^{\delta+})$	Hydrogen
	4 $C = O^{\delta-}$	$K44(N^+H_3)$	Ion-polar
Binding site 7 Affinity: −3.6 kcal/mol	A PO_4^-	$K12(N^+H_3)$	Ionic
	B PO_4^-	$L6(NH_{pb}^{\delta+})$	Ion-polar

	2 $C = O^{\delta-}$	K12($N^{+}H_3$)	Ion-polar
	3 $C = O^{\delta-}$	K12($N^{+}H_3$) C38($NH_{pb}^{\delta+}$)	Ion-polar, hydrogen
Binding site 8<br>Affinity: −3.5 kcal/mol	B PO_4^{-}	Y51($OH^{\delta+}$)	Ion-polar
	1 $C = O^{\delta-}$	K35($N^{+}H_3$)	Ion-polar
	2 $C = O^{\delta-}$	K35($N^{+}H_3$)	Ion-polar
	3 $C = O^{\delta-}$	Y51($OH^{\delta+}$)	Hydrogen
Binding site 9<br>Affinity: −3.5 kcal/mol	A PO_4^{-}	R36(= $N^{+}H_2$) R36($NH_2^{\delta+}$)	Ionic, ion-polar
	$CO^{\delta-}$	R36($NH_2^{\delta+}$)	Hydrogen
	4 $CO^{\delta-}C$	R58($NH_2^{\delta+}$)	Hydrogen

Table S2. Summary of the charged and polar groups of amino acid residues (a.a.r.) in the VII4 binding sites that interact with polar head groups of complete CL molecule. Hypothetical binding sites in VII4 that bind to the charged and polar groups of CL as determined by AutoDock modeling. Designation of PO^{4-} groups and carbon atoms in $CO^{\delta-}$, $CO^{\delta-}C$ and $C = O^{\delta-}$ in CL polar head is shown in Figure S1. Pb in $NH_{pb}^{\delta+}$ denotes a peptide bond.

Site number	CL polar group	1CDT a.a.r. polar group	Bond type
Binding site 1 Affinity: −4.2 kcal/mol	A PO_4^-	R58($NH_2^{\delta+}$)	Ion-polar
	1 $C = O^{\delta-}$	K23(N^+H_3) R36($NH_2^{\delta+}$)	Ion-polar, Hydrogen
	3 $C = O^{\delta-}$	I9($NH_{pb}^{\delta+}$)	hydrogen
Binding site 2 Affinity: −4.2 kcal/mol	$CO^{\delta-}$	R58($NH_2^{\delta+}$) R58(= N^+H_2)	Hydrogen, ion-polar
	3 $CO^{\delta-}C$	R36($NH_2^{\delta+}$)	Hydrogen
	3 $C = O^{\delta-}$	R36(= N^+H_2)	Ion-polar
Binding site 3 Affinity; −4.1 kcal/mol	A PO_4^-	K23(N^+H_3) R36($NH_2^{\delta+}$)	Ionic, Ion-polar
	B PO_4^-	K23(N^+H_3)	Ionic
	3 $CO^{\delta-}C$	R36($NH_2^{\delta+}$)	Hydrogen
Binding site 4 Affinity: −4.0 kcal/mol	A PO_4^-	K35(N^+H_3)	Ionic
	B PO_4^-	L6($NH_{pb}^{\delta+}$) K12(N^+H_3)	Ion-polar, ionic
	$CO^{\delta-}$	Y22($OH^{\delta+}$)	Hydrogen
	1 $C = O^{\delta-}$	K18(N^+H_3)	Ion-polar
Binding site 5 Affinity: −3.9 kcal/mol	3 $C = O^{\delta-}$	C42($NH_{pb}^{\delta+}$)	Hydrogen
Binding site 6 Affinity: −3.9 kcal/mol	A PO_4^-	K12(N^+H_3) R36($NH_2^{\delta+}$)	Ionic, Ion-polar
	2 $C = O^{\delta-}$	R36(= N^+H_2)	Ion-polar
	3 $C = O^{\delta-}$	I9($NH_{pb}^{\delta+}$)	hydrogen
Binding site 7 Affinity: −3.8 kcal/mol	A PO_4^-	R36(= N^+H_2) R36($NH_2^{\delta+}$) K23(N^+H_3)	Ionic, Ion-polar, ionic
Binding site 8 Affinity: −3.8 kcal/mol	A PO_4^-	R36($NH_2^{\delta+}$)	Ion-polar
	2 $CO^{\delta-}C$	R36(= N^+H_2) R36($NH_2^{\delta+}$)	Ion-polar, hydrogen
Binding site 9 Affinity: −3.8 kcal/mol	A PO_4^-	I9($NH_{pb}^{\delta+}$)	Ion-polar
	B PO_4^-	K12(N^+H_3)	Ionic
	$CO^{\delta-}$	L6($NH_{pb}^{\delta+}$)	Hydrogen
	3 $C = O^{\delta-}$	R36($NH_{pb}^{\delta+}$)	Hydrogen

Table S3. Summary of the charged and polar groups of amino acid residues (a.a.r.) in the VII4 binding sites that interact with polar head groups of truncated PC. Hypothetical binding sites in VII4 that bind to the charged and polar groups of PC as determined by AutoDock modeling. Designation of carbon atoms in $CO^{\delta-}C$ and $C = O^{\delta-}$ in PC polar head is shown in Figure S1. Pb in $NH_{pb}^{\delta+}$ denotes a peptide bond.

Site number	PC polar group	1CDT a.a.r. polar group	Bond type
Binding site 1 Affinity: −3.9 kcal/mol	PO_4^-	K12(N^+H_3) C38 ($NH_{pb}^{\delta+}$)	Ionic, Ion-polar
	1 $CO^{\delta-}C$	K12(N^+H_3)	Ion-polar
	1 $C = O^{\delta-}$	L6($NH_{pb}^{\delta+}$)	Hydrogen
Binding site 2 Affinity: −3.9 kcal/mol	PO_4^-	K12(N^+H_3)	Ionic
	1 $C = O^{\delta-}$	R36 ($NH_{pb}^{\delta+}$)	Hydrogen
	2 $C = O^{\delta-}$	K12(N^+H_3) C38($NH_{pb}^{\delta+}$)	Ion-polar, hydrogen
Binding site 3 Affinity: −3.7 kcal/mol	PO_4^-	K12(N^+H_3)	Ionic
	1 $C = O^{\delta-}$	L6($NH_{pb}^{\delta+}$)	Hydrogen
	2 $C = O^{\delta-}$	K12(N^+H_3) C38($NH_{pb}^{\delta+}$)	Ion-polar, hydrogen
Binding site 4	PO_4^-	K18(N^+H_3) N40($NH_2^{\delta+}$)	Ionic, ion-polar
Affinity: −3.7 kcal/mol	1 $C = O^{\delta-}$	K35(N^+H_3)	Ion-polar
Binding site 5	PO_4^-	K12(N^+H_3) C38($NH_{pb}^{\delta+}$)	Ionic, ion-polar
Affinity: −3.6 kcal/mol	1 $C = O^{\delta-}$	K12(N^+H_3)	Ion-polar
Binding site 6 Affinity: −3.5 kcal/mol	PO_4^-	K12(N^+H_3) C38($NH_{pb}^{\delta+}$)	Ionic, ion-polar
Binding site 7 Affinity: −3.4 kcal/mol	PO_4^-	I7($NH_{pb}^{\delta+}$) L6($NH_{pb}^{\delta+}$)	Ion-polar
	1 $C = O^{\delta-}$	R36($NH_2^{\delta+}$)	Hydrogen
	2 $C = O^{\delta-}$	K12(N^+H_3)	Ion-polar
Binding site 8 Affinity: −3.4 kcal/mol	PO_4^-	R58($NH_2^{\delta+}$)	Ion-polar
	2 $CO^{\delta-}C$	R58($NH_2^{\delta+}$) R58($= N^+H_2$)	Hydrogen, ion-polar
Binding site 9 Affinity: −3.4 kcal/mol	PO_4^-	R36($= N^+H_2$) R36($NH_2^{\delta+}$)	Ionic, ion-polar

Table S4. Summary of the charged and polar groups of amino acid residues (a.a.r.) in the VII4 binding sites that interact with polar head groups of complete PC. Hypothetical binding sites in VII4 that bind to the charged and polar groups of PC as determined by AutoDock modeling. Designation of carbon atoms in $CO^{\delta-}C$ and $C = O^{\delta-}$ in PC polar head is shown in Figure S1. Pb in $NH_{pb}^{\delta+}$ denotes a peptide bond.

Site number	PC polar group	1CDT a.a.r. polar group	Bond type
Binding site 1 Affinity: −4.4 kcal/mol	PO_4^-	K12(N^+H_3) K18(N^+H_3) C38 ($NH_{pb}^{\delta+}$)	Ionic, ionic, ion-polar
	2 $CO^{\delta-}C$	K35(N^+H_3)	Ion-polar
Binding site 2	PO_4^-	Y51($OH^{\delta+}$)	Ion-polar
Affinity: −4.3	2 $CO^{\delta-}C$	K35(N^+H_3)	Ion-polar
kcal/mol	1 $C = O^{\delta-}$	K35(N^+H_3)	Ion-polar
Binding site 3 Affinity: −4.2 kcal/mol	PO_4^-	R36($= N^+H_2$) R36($NH_2^{\delta+}$)	Ionic, ion-polar
Binding site 4	PO_4^-	K35(N^+H_3)	Ionic
Affinity: −4.1 kcal/mol	2 $CO^{\delta-}C$	K35(N^+H_3)	Ion-polar
Binding site 5	PO_4^-	R36($NH_2^{\delta+}$)	Ion-polar

Affinity −4.1 kcal/mol	2 $CO^{\delta-}C$	R36(= $N^{+}H_2$)	Ion-polar
Binding site 6 Affinity −4.0 kcal/mol	1 C = $O^{\delta-}$	K44($N^{+}H_3$)	Ion-polar
Binding site 7 Affinity: −4.0 kcal/mol	PO_4^{-}	S46($OH^{\delta+}$)	Ion-polar
Binding site 8 Affinity: −4.0 kcal/mol	PO_4^{-}	K50($N^{+}H_3$)	Ionic
	2 $CO^{\delta-}C$	N45($NH_{pb}^{\delta+}$)	Hydrogen
Binding site 9 Affinity: −4.0 kcal/mol	PO_4^{-}	K29($N^{+}H_3$)	Ionic
	2 $CO^{\delta-}C$	K29($N^{+}H_3$)	Ion-polar

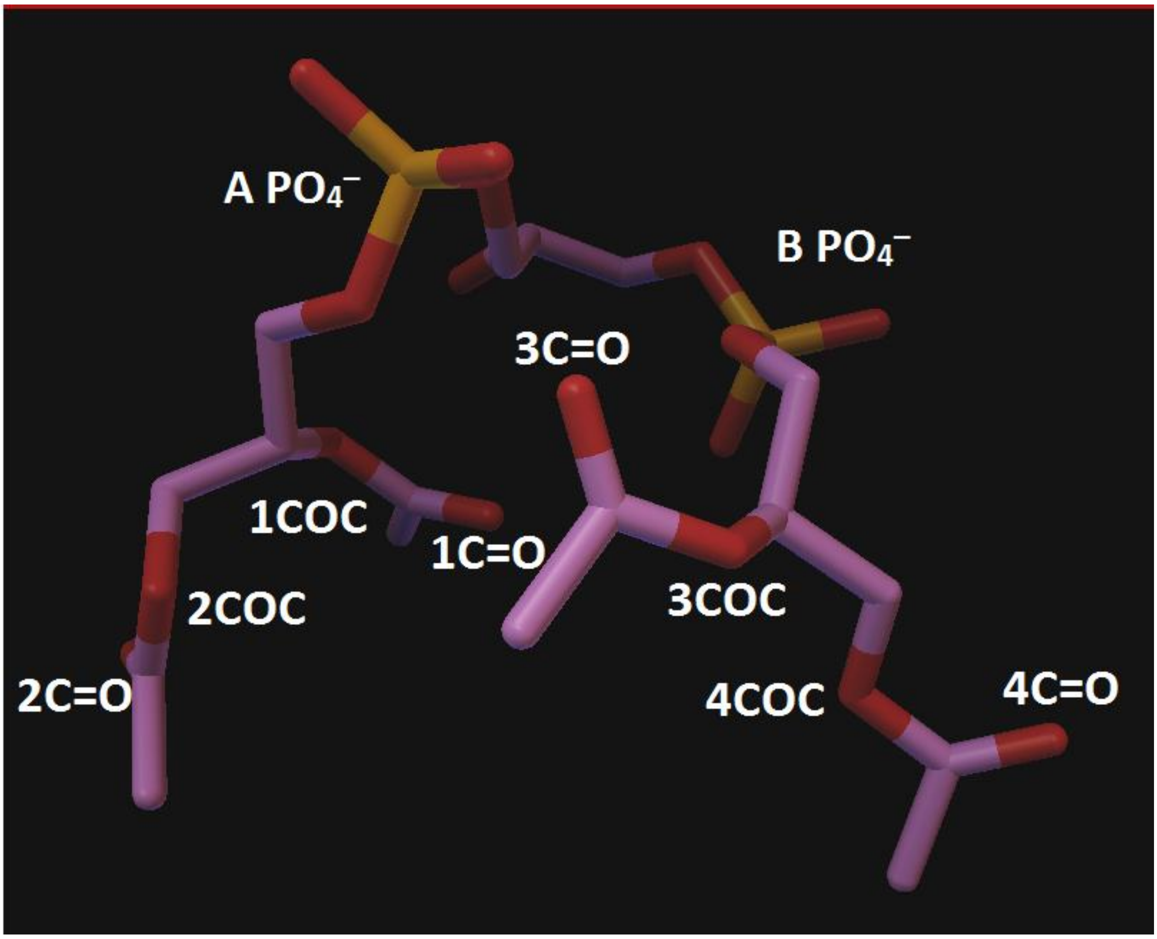

Figure S1. The marking of the charged and polar groups in the CL polar head.

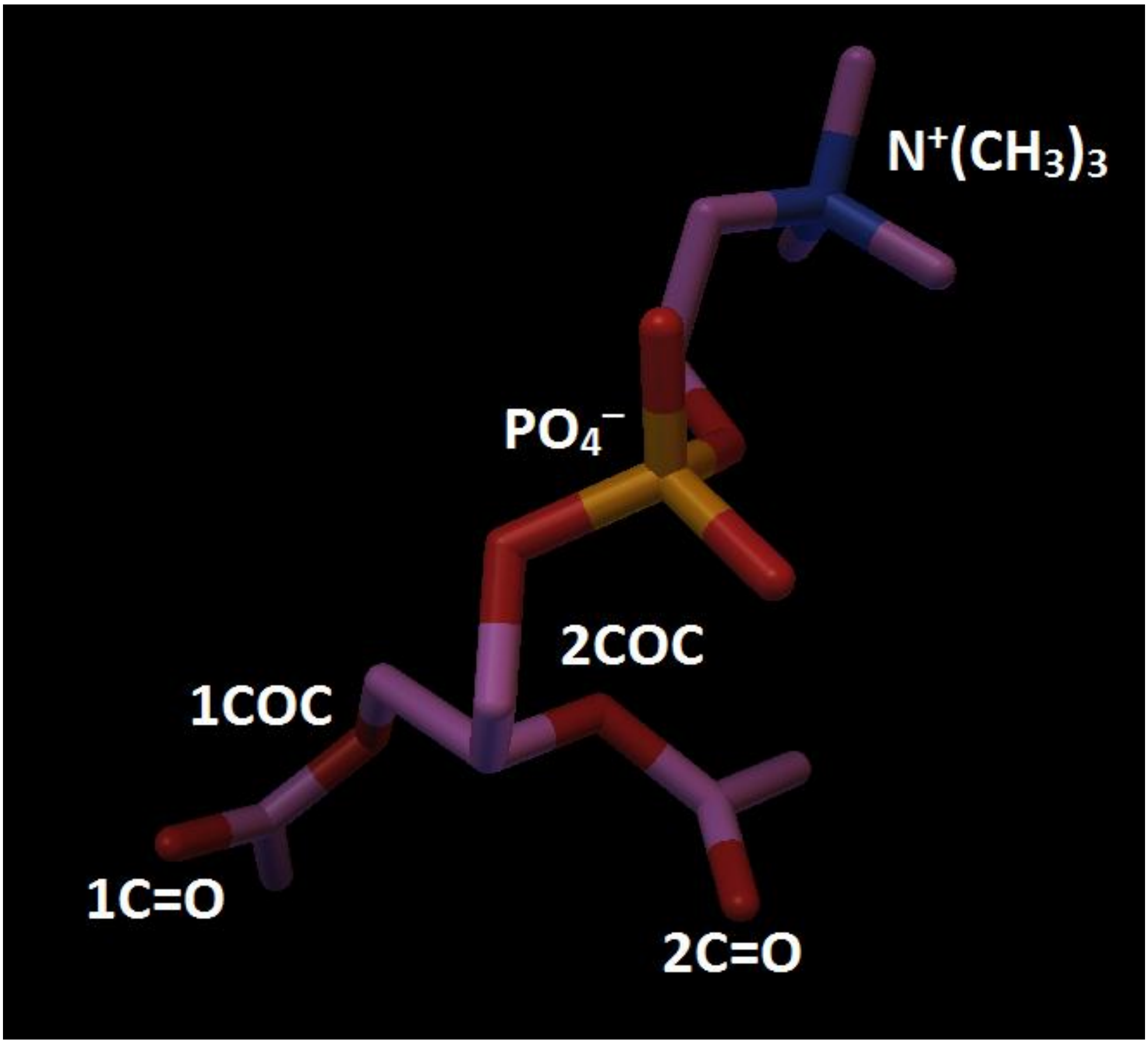

Figure S2. The marking of the charged and polar groups in the PC polar head.
